# Fitness Effects of Phenotypic Mutations at Proteome-Scale Reveal Optimality of Translation Machinery

**DOI:** 10.1093/molbev/msae048

**Published:** 2024-02-29

**Authors:** Cedric Landerer, Jonas Poehls, Agnes Toth-Petroczy

**Affiliations:** Max Planck Institute of Molecular Cell Biology and Genetics, 01307 Dresden, Germany; Center for Systems Biology Dresden, 01307 Dresden, Germany; Max Planck Institute of Molecular Cell Biology and Genetics, 01307 Dresden, Germany; Center for Systems Biology Dresden, 01307 Dresden, Germany; Max Planck Institute of Molecular Cell Biology and Genetics, 01307 Dresden, Germany; Center for Systems Biology Dresden, 01307 Dresden, Germany; Cluster of Excellence Physics of Life, TU Dresden, 01062 Dresden, Germany

**Keywords:** translation error landscape, amino acid misincorporation, translation fidelity, population genetics, mass spectrometry

## Abstract

Errors in protein translation can lead to non-genetic, phenotypic mutations, including amino acid misincorporations. While phenotypic mutations can increase protein diversity, the systematic characterization of their proteome-wide frequencies and their evolutionary impact has been lacking. Here, we developed a mechanistic model of translation errors to investigate how selection acts on protein populations produced by amino acid misincorporations. We fitted the model to empirical observations of misincorporations obtained from over a hundred mass spectrometry datasets of *E. coli* and *S. cerevisiae*. We found that on average 20% to 23% of proteins synthesized in the cell are expected to harbor at least one amino acid misincorporation, and that deleterious misincorporations are less likely to occur. Combining misincorporation probabilities and the estimated fitness effects of amino acid substitutions in a population genetics framework, we found 74% of mistranslation events in *E. coli* and 94% in *S. cerevisiae* to be neutral. We further show that the set of available synonymous tRNAs is subject to evolutionary pressure, as the presence of missing tRNAs would increase codon–anticodon cross-reactivity and misincorporation error rates. Overall, we find that the translation machinery is likely optimal in *E. coli* and *S. cerevisiae* and that both local solutions at the level of codons and a global solution such as the tRNA pool can mitigate the impact of translation errors. We provide a framework to study the evolutionary impact of codon-specific translation errors and a method for their proteome-wide detection across organisms and conditions.

## Introduction

Genetic information is processed with a high fidelity that is essential for cellular life, yet the processing is not exact. Despite the importance of proteins, their production is an error-prone process with errors occurring far more frequently than genetic mutations (Romero Romero et al. 2022). The mutations that result from these errors in transcription and translation are collectively termed phenotypic mutations (Bürger et al. 2006; Goldsmith and Tawfik 2009; Yanagida et al. 2015; Bratulic et al. 2017). Most phenotypic mutations are likely stochastic events without no function, as was argued for stop codon readthrough errors (Li and Zhang 2019). Nevertheless, they contribute to protein diversity and can have adaptive function, such as for certain ribosomal frameshifts and stop codon readthrough in viruses, bacteria and yeast (Romero Romero et al. 2022). The impact of amino acid misincorporations on protein diversity is less well understood. While most amino acid misincorporations are expected to be neutral or have a deleterious effect, e.g. via misfolding or aggregation, and induce proteotoxic stress in the cell (Drummond and Wilke 2008), some can be beneficial and lead to altered functionality (Miranda et al. 2013).

Amino acid misincorporations can result from either transcription or translation errors, and the latter include both charging a tRNA with the wrong amino acid (mischarging) and the ribosome binding the wrong tRNA (mispairing) (Ibba and Söll 1999). Most misincorporations are the result of mispairing between codons and tRNA anti-codons. Specifically, Kramer and Farabaugh observed experimentally (Kramer and Farabaugh 2007) and Mordret et al. confirmed on a proteome scale that the competition of cognate (Watson-Crick or wobble pairing, correct amino acid) and near-cognate (one mismatch, different amino acid) tRNAs determines the mistranslation rate of a codon (Mordret et al. 2019).

While we have an understanding of how amino acid substitutions impact protein fitness, we do not know the impact of amino acid misincorporations. There are several experimental methods (e.g. Deep Mutational Scans, collected in MAVE database; Esposito et al. 2019) and computational tools (e.g. Variant Effect Predictors; Livesey and Marsh 2020), which allow exploration of the fitness landscape of a protein, i.e. the effects of all potential substitutions. However, the evolutionary relevance of amino acid misincorporations depends not only on the effect of the substitutions but also on their overall occurrence, i.e. the error rate. Therefore, it is crucial to have proteome-wide estimates of misincorporations in order to study the error-aware fitness landscape and its impact on protein evolution.

Transcriptome-wide transcription error estimates based on RNA-seq data range from $∼10^{-6}$ nucleotide misincorporation per codon in *C. elegans* (Gout et al. 2013), *E. coli*, *B. subtilis*, and *A. tumefaciens* (Li and Lynch 2020) to $∼10^{-5}$ in *S. cerevisiae* (Gout et al. 2017; Reid-Bayliss and Loeb 2017) and *M. florum* (Li and Lynch 2020). Translation error rates are difficult to measure proteome-wide and are instead often measured for individual reporter constructs. These measurements resulted in an estimate of $∼10^{-3}$ amino acid misincorporation per codon (Loftfield 1963; Loftfield and Vanderjagt 1972; Gromadski and Rodnina 2004; Kramer and Farabaugh 2007; Drummond and Wilke 2009). So far, only one study measured amino acid misincorporation rates proteome-wide and found stark differences between codons, with average error rates ranging from $∼10^{-4}$ to $∼10^{-3}$ amino acid misincorporation per codon in *E. coli* (Mordret et al. 2019). Thus, overall amino acid misincorporations are thought to be frequent.

How do organisms cope with such high error rates in protein production? Experiments showed that organisms can employ different strategies to minimize the impact of higher than endogenous error rates of protein production: they may either regulate the level of protein production or accumulate stabilizing mutations to buffer the deleterious effects of phenotypic mutations as both shown in different experiments (Goldsmith and Tawfik 2009; Bratulic et al. 2015). Despite these observations, it is still unknown if selection to increase endogenous translation fidelity is strong enough to overcome adverse effects by mutations or potential costs associated with increased fidelity. Translation fidelity can be increased via (1) a global solution that impacts the whole proteome or (2) local solutions on a gene by gene or amino acid site by site basis (Kurland 1992). A global solution might mean to evolve a higher fidelity ribosome, e.g. by more efficient kinetic proofreading (Hopfield 1974) or conformational changes of the ribosomes’ decoding center (Ibba and Söll 1999). Local solutions can be achieved by optimizing synonymous codon usage of individual sites for higher translation fidelity (Sun and Zhang 2022). There is evidence for local solutions: Error rates are lower for conserved and highly expressed sites, indicating selection for high translation fidelity of important sites and proteins (Mordret et al. 2019).

While experimental measurements provide data on select genes only or on a snapshot of proteome-wide amino acid misincorporation, theoretical models can provide error estimates for all sites. Previous models calculated error rates from general kinetic ribosome parameters and tRNA abundances (Fluitt et al. 2007; Shah and Gilchrist 2010). Since these models distinguish between tRNAs solely based on their abundance and their classification as (near-) cognate or non-cognate, they cannot provide any insights into specific codon/tRNA interactions.

Here, we combine the advantages of mining proteome-wide data and mechanistic modeling to model the complete error landscape of *S. cerevisiae* and *E. coli* in order to study the impact of misincorporations on the fitness landscape and the evolution of translation fidelity. We developed a mechanistic model of translation errors (mechanistic Translation Error Landscape, mTEL) with parameters fitted to proteome-wide amino acid misincorporation data from hundreds of mass spectrometry datasets of *S. cerevisiae* and *E. coli* proteins obtained from the PRIDE repository (Jones and Côté 2008, Perez-Riverol et al. 2018, 2022) (empirical Translation Error Landscape, eTEL). By performing *in silico* experiments, we quantify the evolutionary impact of amino acid misincorporations. We found that deleterious misincorporations have lower error probability, indicating selection against deleterious misincorporations. Combining the estimated error probabilities and fitness effects of amino acid misincorporations revealed that at the majority of sites, amino acid misincorporations are neutral. Overall, only a small fraction of proteins exhibits a fitness burden due to their error-prone translation. Therefore, it is likely that for neither species the efficacy of selection is strong enough to further increase global translation fidelity. Here, based on data of proteome-wide translation errors, we detect “local” solutions to amino acid misincorporations such as codon choice and describe how “global” solutions such as the presence/absence of tRNA species can increase/decrease translation fidelity. In summary, we present a data-driven, proteome-wide assessment of translation errors across hundreds of datasets and provide a framework rooted in population genetics to estimate the selection organisms face on translation fidelity.

## Results

### Proteome-wide Amino Acid Misincorporations Detected in Hundreds of Mass Spectrometry Datasets

While amino acid misincorporation have been explored individually for decades via reporter constructs (Gromadski and Rodnina 2004; Kramer and Farabaugh 2007; Drummond and Wilke 2009), proteome-wide identification of amino acid misincorporations is only achievable with mass spectrometry. So far, only one study identified amino acid misincorporations proteome-wide, based on a single dataset (Mordret et al. 2019).

**Fig. 1.**
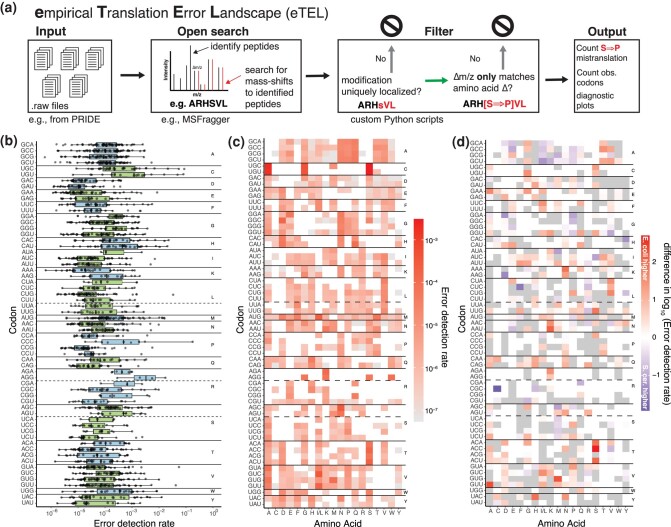
Detecting translation errors in mass spectrometry data. a) High-throughput pipeline for the detection of the eTEL based on existing mass spectrometry datasets. Raw files are processed using MSFragger's open search, and identified peptides are FDR controlled (0.05). Modified peptides are only retained if the modification can be uniquely localized and the mass difference can only be explained by an amino acid misincorporation. Codon and peptide counts are obtained over all peptides detected. b) Distribution of error detection rates across datasets (n = 80) shown for each codon grouped by the encoded amino acid. Note that not all substitutions are present in all datasets. Codon-specific error detection rates for all codons vary greatly between synonymous codons, indicating large differences in fidelity between codons. Similarly, a large variation in the detection rate between datasets highlights the intrinsic noise in mass spectrometry data. c) Heatmap of codon-specific error detection rates shown for each target amino acid identified across 80 datasets of *E. coli*. Synonymous codons often show the same errors (e.g. GCN to N, P, and Q); however, many errors are detected only at individual codons (e.g. UCC to Y). d) The individual error detection rates of *E. coli* and *S. cerevisiae* can differ substantially. For example, CGC to C misincorporations are much more common in *S. cerevisiae* (blue), while ACC to S is more common in *E. coli* (red). Many differences could not be calculated as an estimate for either *E. coli* or *S. cerevisiae* was missing (gray); white indicates the absence of both estimates.

However, mass spectrometry datasets have large variation in the identified peptides and their frequencies, due to differences in biological and technical conditions. In order to capture this variation, it is important to consider a large number of datasets. To achieve this, we developed a high-throughput pipeline based on MSFragger's (Kong et al. 2017) open search algorithm (Yu et al. 2020) (Fig. 1a). The pipeline identifies, post-processes, and filters both unmodified and modified peptides. Modified peptides are those that show a mass shift compared to the canonical protein sequence. For the identification of amino acid substitutions in modified peptides, we only considered those peptides that were also identified as unmodified and were uniquely assigned to a protein. We further used stringent quality filters to avoid the misidentification of mass-artefacts and post-translational modifications (PTMs) as amino acid misincorporations (see Methods for details and Fig. 1a). We used this pipeline to re-analyze hundreds of mass spectrometry datasets of *S. cerevisiae* and *E. coli* from the PRIDE repository (Jones and Côté 2008; Perez-Riverol et al. 2018, 2022) (supplementary Data S1, Supplementary Material online). We refer to experimentally detected amino acid substitutions as the eTEL.

In total, we identified 58,990 amino acid misincorporations across 80 datasets for *E. coli* (supplementary fig. S1a, Supplementary Material online), and 17,778 amino acid misincorporations across 72 datasets for *S. cerevisiae* (supplementary fig. S1b, Data S1, Supplementary Material online). Overall, the number of the detected specific amino acid misincorporations is correlated with the number of identified peptides per dataset on the logarithmic scale (two-sided Wald-test, Pearson's $R^{2}=0.32$, $p<4.4\;\;\times \;\;10^{-8}$, n=80,  supplementary fig. S1c, Supplementary Material online, *E. coli*, Pearson's $R^{2}=0.49$, $p<8.4\;\;\times \;10^{-12}$, n=72; supplementary fig. S1d, Supplementary Material online, *S. cerevisiae*), indicating that the number of identifiable amino acid misincorporations is correlated with the depth of a given mass spectrometry experiment.

Our high-throughput error detection pipeline revealed that amino acid misincorporations occur across a large variety of datasets and experimental conditions, and the deeper the mass spectrometry data is, i.e. has a higher coverage (more detected peptides), the more misincorporations can be identified.

### Codon-specific Error Detection Rates Vary by Orders of Magnitude

Quantifying the error rate is non-trivial based on label-free mass spectrometry measurements. Previously, error rates were calculated as the ratio of MS1 peak heights (in the mass spectrometry chromatogram) of the corresponding mistranslated (containing an amino acid substitution) and correct peptides (Mordret et al. 2019). Here, we deliberately decided to use an alternative approach that is not dependent on the mass spectrometry chromatograms, which are affected by the properties of the peptides and the dynamic ranges of the instruments, which become an issue when the intensities are orders of magnitude apart (see Discussion) (Cech and Enke 2000; Arike et al. 2012). Instead, we are using all the detected peptides of eTEL with and without amino acid misincorporations to aggregate the codon counts for each dataset. We estimate the error rate of a codon *i* by dividing the number of times codon *i* was observed to be mistranslated by the total number of times codon *i* was observed in any peptide (Fig. 1b, supplementary fig. S2a, Supplementary Material online). Since these error estimates are limited by biases in detection as detailed below, we will use the term *error detection rate*. The protein-specific error detection rate was calculated as the ratio of erroneous and total number of peptides associated with a protein. The dependency of the error detection rate on the number and composition of the identified peptides likely results in an underestimate, as we are bound to miss the rarest of misincorporation events; affecting the numerator (mistranslated codons) much stronger than the denominator (correctly translated codons). In both species, we observe a clear bias toward highly abundant proteins harboring more amino acid misincorporations ($\rho =0.59,\;p<2.2\;\times \;10^{-16}$  supplementary fig. S3a, Supplementary Material online; *E. coli*; ρ=0.45,  $p<2.2\;\times \;10^{-16}$, supplementary fig. S3b, Supplementary Material online, *S. cerevisiae*) in agreement with previous observations (Mordret et al. 2019). While the error detection rate estimates for *E. coli* follow this same trend ($\rho =0.16,\;p<1.1\;\times \;10^{-8}$  supplementary fig. S3c, Supplementary Material online), *S. cerevisiae* error detection rates show a slight negative correlation with protein abundance ($\rho =-0.11,\;p=2\times 10^{-4}$  supplementary fig. S3d, Supplementary Material online). In general, amino acid misincorporations in low-abundance proteins are underrepresented in all examined datasets (supplementary fig. S3e, Supplementary Material online, *E. coli*; supplementary fig. S3f, Supplementary Material online, *S. cerevisiae*).

Error detection rates of codons vary by orders of magnitude between datasets (Fig. 1b, supplementary Data S2, Supplementary Material online). The valine codon GUA and the histidine codon CAC show the highest variation among datasets, while the arginine codons AGA and CGA have the lowest variation (supplementary fig. S4a, Supplementary Material online, *E. coli*; supplementary fig. S4b, Supplementary Material online, *S. cerevisiae*). The ability to quantify the variation in amino acid misincorporation rates is a clear advantage of proteomics approaches over rate quantification via individual constructs. Importantly, the variation that we observe is a property of the mass spectrometry datasets and not a result of our data processing pipeline. Overall, the large differences in the error estimates from different datasets highlight the need for aggregating data systematically and to present error rates along with their variations across measurements.

### A Comprehensive Translation Error Landscape at the Codon Level

A wide variety of tRNAs can be wrongly incorporated at any codon, leading to different amino acid misincorporations. While each dataset provides a snapshot of amino acid misincorporations, to obtain a comprehensive translation error landscape at the codon level, detected amino acid misincorporations were pooled across all datasets and plotted as a codon to amino acid misincorporation matrix (Fig. 1c, *E. coli,*  supplementary fig. S2b, Supplementary Material online, *S. cerevisiae*). Each entry represents the error detection rate, i.e. the number of times a codon was observed to be wrongly translated with a given amino acid divided by the total number of observations for this codon. Leucine and isoleucine are treated as the same error because they cannot be distinguished in mass spectrometry data due to their identical masses.

This matrix allows us to see and compare the patterns of misincorporation for each codon. We can identify hotspots of substitutions, such as UGU (cysteine) to serine or AUG (methionine) to (iso)leucine (supplementary Data S2, Supplementary Material online), while many other substitutions are never observed. The number of unique amino acids misincorporated at a given codon also differs greatly between codons. For example, for AUG (methionine), we detect 17 different amino acids, while AGA (arginine) misincorporates only lysine in *E. coli* (Fig. 1c, supplementary fig. S4a, Supplementary Material online). This indicates that a codon's overall error rate does not necessarily reflect the variation in the amino acids misincorporated. This analysis may be biased, however, since the number of synonymous codons varies between amino acids (e.g. methionine having one and arginine having six) and that certain misincorporations are indistinguishable from other PTMs and cannot be detected (supplementary Data S3, Supplementary Material online). An important characteristic of misincorporation types is the number and position of mismatches they require. Although we see all types of nucleotide mismatches (supplementary fig. S5a, Supplementary Material online, *E. coli*; supplementary fig. S5b, Supplementary Material online, *S. cerevisiae*), most observed mismatches involve the first codon position, either alone or in combination with another position. Misincorporations caused by a single mismatch are most frequently observed at the second codon position (supplementary fig. S5c, Supplementary Material online, *E. coli*; supplementary fig. S5d, Supplementary Material online, *S. cerevisiae*). Due to the degeneracy of the genetic code, mismatch at the third (wobble) codon position—being least frequent—often leads to synonymous change which does not alter the amino acid.

Further, we can observe differences in the error hotspots between *E. coli* and *S. cerevisiae*. For example, CGC (arginine) to cysteine misincorporations are much more likely in *S. cerevisiae* than *E. coli*, and the opposite is true for ACC (tyrosine) to serine or GUU (valine) to alanine (Fig. 1d). Overall, we provide a comprehensive error landscape as a codon to amino acid matrix. Based on a large number of mass spectrometry datasets, we estimate error rates for the majority of possible amino acid misincorporations and identify hotspots of amino acid misincorporation errors.

### Mechanistic Modeling of tRNA Incorporation Allows Exploration of the Complete Translation Error Landscape

In order to explore the complete translation error landscape and perform *in silico* experiments probing the mechanisms of translation fidelity, we developed a mechanistic model of amino acid misincorporations. We refer to our model as mTEL.

Translation fidelity is ensured via structural and kinetic interactions between the ribosome, its co-factors, and the tRNA (Blanchard et al. 2004; Budkevich et al. 2011). These interactions serve as proofreading mechanisms giving the ribosome the ability to avoid amino acid misincorporations by rejecting non-synonymous tRNAs. However, if the correct tRNA does not arrive at the ribosome in a timely manner, the ribosome will stall due to an empty A-site (Schuller and Green 2018). Stalling can lead to frameshifts or to the incorporation of non-synonymous tRNAs, which results in an amino acid misincorporation (Schuller and Green 2018). Accordingly, the relative abundance of cognate and near-cognate tRNAs was suggested to be the major determinant of codon mistranslation rates (Kramer and Farabaugh 2007; Mordret et al. 2019).

**Fig. 2.**
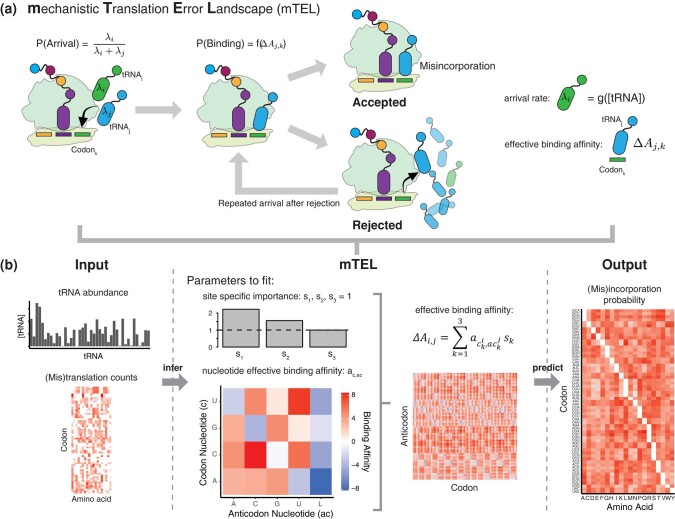
Modeling translation errors based on tRNA competition. a) The mTEL model is a model of tRNA misincorporations at steady state. The probability of tRNA incorporation is controlled by competition for arrival at the ribosome determined by tRNA abundance and an effective binding affinity between codon and anticodon. Arrival of tRNAs is modeled as exponential waiting times. The probability of tRNA_i_ arriving before a competing tRNA_j_ is defined as the ratio between expected arrival rates. Differences in the probability to arrive before a competitor can be substantial (supplementary fig. S6a, Supplementary Material online). Effective binding affinities between codon and anticodons determine the probability a tRNA is accepted/rejected and is estimated from observed amino acid misincorporations. However, differences in tRNA abundances can lead to cases were a tRNA arrives multiple times before the correct tRNA will arrive, leading to multiple binding attempts. b) mTEL uses tRNA abundance estimates from RNAseq data and mistranslation counts derived from the eTEL pipeline to predict amino acid misincorporation probabilities. First, tRNA abundances are used to calculate expected tRNA arrival rates, based on diffusion through the cell (Fluitt et al. 2007). Second, Nucleotide interaction parameters (20 for *E. coli* and 16 for *S. cerevisiae*) and two site-specific importance parameters are inferred (shown for *E. coli*; L indicates lysidine, found in bacteria tRNA) from the observed amino acid misincorporations (using MCMC, see Methods). Next, we calculate the effective binding affinity of all pairs of codon *i* (*c_i_*) and anticodon *j* (*c_j_*) (61 × 61 parameters) as a linear combination of the estimated parameters for all three nucleotide positions (*k*) (see Methods for details). We call our binding affinity “effective” as it absorbs any proofreading or conformational changes contributing to translation fidelity.

Therefore, we modeled the probability of tRNA incorporation as a function of tRNA arrival rates and of codon/anticodon binding affinity (Fig. 2, supplementary fig. S6a, Supplementary Material online, see Methods). In short, we considered tRNA incorporation as a multistep process: First, a tRNA has to arrive at the ribosome (Eq. 1), and second, it has to bind to the codon and pass proofreading by the ribosome (Eq. 2). For tRNA arrival, we considered the probability of two cases: that a given tRNA arrives first or that other tRNAs arrive beforehand and have to be rejected. The expected number of competing tRNA arrival events before the focal tRNA differs for low, medium, and high abundance codons (supplementary fig. S6b, Supplementary Material online). We assumed that tRNAs move through a cell by diffusion only, making expected arrival times of tRNAs at the ribosome proportional to their abundance (Fluitt et al. 2007; Weinberg et al. 2016). Assuming exponentially distributed arrival times, the probability that tRNA *i* will arrive before tRNA *j* is given by their expected arrival times *λ* (supplementary fig. S6a, Supplementary Material online):


(1) $$P_{a}^{i,j}=\frac{\lambda_{i}}{\lambda_{i}+\lambda_{j}}$$

This relationship can be extended to all potential subsets of tRNAs, e.g. synonymous and non-synonymous tRNAs by defining $\lambda_{i}$ and $\lambda_{j}$ as the sum of the arrival rates over the respective sets.

Once a tRNA arrives at the ribosome, its anticodon will attempt to bind the codon. We modeled the codon/anticodon binding affinity as an *effective binding affinity*, a unit-less parameter, describing not only binding strength but also absorbing any additional linear effects of tRNA recognition by the ribosome, such as induced conformation changes of the ribosome (Ibba and Söll 1999) and kinetic proofreading (Hopfield 1974). We described codon/anticodon affinities as a linear combination of nucleotide affinities and position specific parameters assuming additivity of the binding affinities (Fig. 2b). This not only reduces the number of parameters to 22 (four by five nucleotide affinities, with lysidine as the fifth anticodon nucleotide, and two position specific parameters; 18 for *S. cerevisiae* without lysidine), but leverages information about nucleotide interaction across multiple sites and vice versa. We calculated the effective binding affinity $\Delta A_{i,j}$ between a codon *i* and an anticodon *j* as the sum over the individual nucleotide interactions of each codon position, weighted by position (Eq. 7; see Methods for details). We then assumed that the probability of binding ($P^{b}$) between codon *i* and anticodon *j* is proportional to their effective binding affinity:


(2) $$P_{b}^{i,\;j}\propto \exp[-\Delta A_{i,\;j}]$$

Further, we assumed that the number of misincorporated amino acids follows a multinomial distribution, where the incorporation probability ($P^{I})$ is a function of tRNA abundance and the effective codon/anticodon binding affinity $(\Delta A_{i,j}$, see Eq. 11).

Using mTEL, we can infer tRNA misincorporation rates of unobserved misincorporations, based by fitting nucleotide and site-specific parameters to observations made with eTEL and provide a general model of the error landscape.

### Nucleotide Interactions Determine Codon/Anticodon-Specific Affinities

We fitted the nucleotide- and position-specific effective binding parameters of mTEL to eTEL, i.e. the observed amino acid misincorporations in the reanalyzed mass spectrometry datasets (supplementary Data S1, Supplementary Material online). The linear combination of nucleotide effective binding affinities and site-specific importance parameters (*s_1_, s_2_, s_3_*) allows to estimate codon/anticodon binding affinities (Eq. 8). As expected, the estimated effective nucleotide affinities show an increased affinity for the canonical Watson-Crick nucleotide pairs (Fig. 2b, supplementary fig. S7a, Supplementary Material online, supplementary table S1, Supplementary Material online). Interestingly, the nucleotide harboring a Lysidine modification used by bacteria (Nilsson and Alexander 2019) to translate the isoleucine codon AUA seems to interact favorably with all nucleotides (Fig. 2c). It may explain the elevated error rate of the Ile codon AUA (mean: $8.25\times 10^{-4}$) canonically translated by the lysidine-modified Ile-tRNA^CAU^ (Fig. 1b).

Overall, the nucleotide effective binding affinity parameters correlate well ($R^{2}=0.78,p=6.15\times 10^{-6},n=16$, supplementary fig. S7b, Supplementary Material online) between the two species, likely due to the high degree of conservation of the translation machinery across the tree of life. It is, however, unclear if differences in parameter estimates between species originate from differences in tRNA availability and abundances can be attributed to different selective pressures and despite high conservation be attributed to evolutionary distance or simply be caused by differences in the detected misincorporations due to technical noise.

### Amino Acid Misincorporations are Common, but Deleterious Ones are Less Likely to Occur

**Fig. 3.**
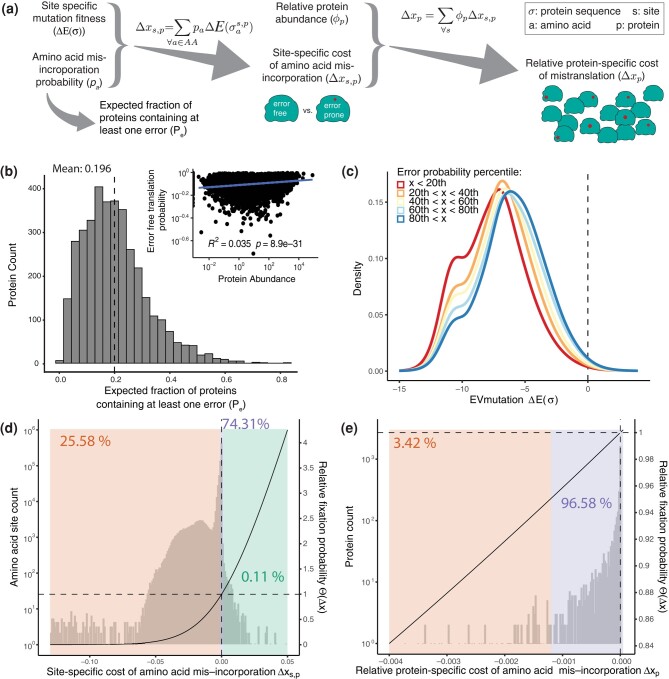
Deleterious amino acid misincorporations are compensated by reduced error rate in *E. coli*. a) Schematic of the calculations of site- ($\Delta x_{s,p}$) and protein- ($\Delta x_{p}$) specific fitness effects from fitness effects calculated by EVmutations (E(σ)) and the fitted amino acid misincorporation probabilities. Amino acid misincorporations are drawn as red shapes on the schematic protein molecules. b) Expected fraction of each protein with at least one amino acid misincorporation. 19.6% of all produced proteins are expected to contain at least one amino acid misincorporation. c) The fitness effects of substitutions were estimated using EVmutations (lower score means increased severity). The predicted severity decreases with increased error probability (binned by error probability from red to blue) (see supplementary table S4, Supplementary Material online), suggesting that the translation machinery prevents strongly deleterious misincorporations. d) Distribution of site-specific costs of amino acid misincorporations in *E. coli* (left y-axis) and the relative fixation probability (right y-axis) an amino acid misincorporation with a given cost has (black curve). The majority (∼74.31%) of amino acid misincorporations are neutral (purple region), a smaller percentage (∼25.58%) are deleterious (orange region), and only a very small fraction (∼0.11%) is considered advantageous (green region). The black curve shows the relative fixation probability, and the dashed lines indicate the shift from decreased (Θ(Δx)<1) to increased relative fixation probability (Θ(Δx)>1) (right axis). Δx<−0.13 was binned together. e) Distribution of protein-specific cost of amino acid misincorporations in *E. coli* (left y-axis) and the relative fixation probability (right y-axis) an amino acid misincorporation with a given cost has (black curve). Fitness burden was calculated as the sum of individual site contributions and weighted by the relative contribution of a protein to the proteome based on protein abundance (Wang et al. 2015). About 3.42% of proteins impose a significant burden due to amino acid misincorporations in *E. coli*.

In order to better understand the effects of translation errors on individual proteins, we explored, using mTEL, how amino acid misincorporations and their effects are distributed across the protein sequence and across the proteome (Fig. 3a). First, we calculated the probability that a protein is translated error free (Eq. 13) and we found that on average 19.6% of *E. coli* proteins (23.4% in *S. cerevisiae*) are expected to contain at least one error (Fig. 3b, supplementary fig. S7c, Supplementary Material online). The probability to translate a protein error-free is, however, not uniformly distributed but varies greatly between proteins and increases slightly with expression level (Fig. 3a inset, supplementary fig. S7c, Supplementary Material online, inset). Differences in the distribution of error-free proteins may be explained by the generally longer proteins in *S. cerevisiae*, given that the error detection rates estimated for *E. coli* and *S. cerevisiae* are similar ($R^{2}=0.42,\;p=1.92\times 10^{-49}$, supplementary fig. S7d, Supplementary Material online). This is further underlined by the observation that the proportion of error-free proteins declines with protein length (supplementary fig. S7e and f, Supplementary Material online).

Given the high proportion of error-containing proteins, it is important to understand the impact amino acid misincorporations have on protein function and fitness. To this end, using EVmutation scores (Hopf et al. 2017) as a proxy, we estimated fitness effects for all possible mutations at a given codon position in the proteome. EVmutation is an unsupervised statistical method for predicting the effects of mutations in any organism from protein sequence σ, based on amino acid alignments. It considers both residue conservation and epistasis (co-evolution) between positions to estimate the relative fitness effect of a mutation compared to a reference amino acid (ΔE(σ)), in our case the correct amino acid. Across 4,584 and 3,081 proteins with sufficient alignment depth in *E. coli* and *S. cerevisiae,* respectively, we calculated in total 26,504,184 and 13,117,068 fitness effects. When we binned the estimated fitness effects by error probability, we found that, both in *E. coli* (Fig. 3b) and in *S. cerevisiae* (supplementary fig. S8a and b, Supplementary Material online), the distribution of fitness effects shifts with increasing error probability (red to blue): error probabilities are lower for more deleterious amino acid misincorporations (supplementary table S2, Supplementary Material online). These conclusions are the same when using Envision (Gray et al. 2018), a supervised method trained on large-scale mutagenesis data, to predict the impact of mutations instead of EVmutation scores (supplementary fig. S8c and d, Supplementary Material online). EVmutation estimates are based on sequence alignments across many species, which represent substitutions between organisms that were subjected to selection on the protein and the organism level. Therefore, EVmutation fitness estimates are confounded by organismal fitness and do not only represent molecular fitness. Envision estimates, however, are closer to molecular fitness effects, since the algorithm was trained on large-scale mutagenesis data where the readouts are mainly linked to protein function. Overall, our findings seem robust to the choice of fitness estimates. Our results highlight the abundance of mistranslated proteins (20% to 23%) and show a tendency to minimize amino acid misincorporation probability where they are the most harmful, indicating potential local solutions to translation errors.

### Few Proteins Contribute to the Fitness Burden of Error-prone Translation

So far, we considered only the fitness difference between a specific misincorporation-containing protein and the error-free (WT) protein. However, the overall fitness effects of errors on the whole protein population depend on the error rate, which determines the fractions of wild-type (error-free) and error-containing protein molecules. Therefore, we combined the probability for a codon *c* at a site *s* to misincorporate a given amino acid *a* ($P_{s(c),a}$) estimated with mTEL with the estimated fitness effects of that amino acid substitution at the given codon site (s) of a protein *P* with sequence *σ* ($\Delta E(\sigma_{a}^{s,\;p})$; Eq. 3), accounting for all possible amino acid misincorporations to calculate the site-specific fitness effects of amino acid misincorporations in each protein *P* ($\Delta x_{s,p}$) relative to a hypothetical error-free translation machinery:


(3) $$\Delta x_{s,\;p}=\underset{\forall a\in AA}{\sum }\;P_{s(c),\;a}\Delta E(\sigma_{a}^{s,\;p})$$

In order to contextualize the site-specific fitness effects of amino acid misincorporations, we estimated the fixation probability of that protein with a given proportion of mistranslations at the focal site. We computed the relative fixation probability (Eq. 14), Θ(Δx) of amino acid misincorporations, based on (Sella and Hirsh 2005) compared to the correctly translated protein (x=0).

There is large uncertainty in the observed error rates as well as in the model estimates since many factors play a role in translation fidelity and consequently the fixation probability beyond the ones considered here. To account for this uncertainty, we introduced nearly neutral mutations inspired by the concepts of nearly neutral theory by Ohta (Ohta 1996; Ohta and Gillespie 1996). We consider here a fitness effect that results in at most a 5% change in fixation probability as neutral. Overall, the relative fixation probability estimates show that at the majority of sites, amino acid misincorporations are (nearly) neutral (defined as the fixation probability being within 5% of a neutral change) in both *E. coli* and *S. cerevisiae* (74.31% and 94.24%, right axes, Fig. 3c; supplementary fig. S8c and d, Supplementary Material online). In contrast, at 25.58% and 5.75% of sites, amino acid misincorporations are deleterious (or even advantageous in 0.11% and 0.01% of cases) in *E. coli* and *S. cerevisiae*, respectively (Fig. 3c; supplementary fig. S8c and d, Supplementary Material online).

We further assessed the global effects of amino acid misincorporations on the entire proteome by summing the fitness estimates over all site-specific estimates for each individual protein (Eq. 4), utilizing the additive properties of the used fitness estimates (Sella and Hirsh 2005). Individual protein fitness effects were then weighted by their relative protein abundance ($\phi_{p}$) from PAXdb (Wang et al. 2015) to estimate their contribution to the total amount of proteins produced as a means to approximate protein importance independent of protein function and environmental factors. Then, we calculated the fitness burden $\Delta x_{p}$ of a given protein's error-prone translation as a sum over all sites *s* within the protein:


(4) $$\Delta x_{p}=\phi_{p}\underset{\forall s}{\sum }\;\Delta x_{s,p}$$

We found that only a small fraction of proteins exhibit a fitness burden due to their error-prone translation on *E. coli* (∼3.42%, Fig. 3d) and on *S. cerevisiae* (0to∼0.07%, supplementary fig. S8e and f, Supplementary Material online).

Since only a small fraction of sites and proteins contribute to the fitness burden, *S. cerevisiae* is unlikely to face strong selection to further increase global translation fidelity (range of relative fixation probability Θ = [0.93 to 1.00], supplementary fig. S8e, Supplementary Material online). *Escherichia coli*, on the other hand, could show a higher efficacy of selection on individual proteins despite smaller Δ*x*, due to its higher effective population size (range of relative fixation probability Θ = [0.84 to 1.00], Fig. 3d). Thus, instead of global increase of translation fidelity, local solutions—like site-specific codon choice—to increase fidelity may be preferred over global solutions which potentially incur high energetic costs.

### Differential Fitness Effects of Amino Acid Misincorporations at Synonymous Codon Sites Offers Local Solutions to Mitigate Errors

**Fig. 4.**
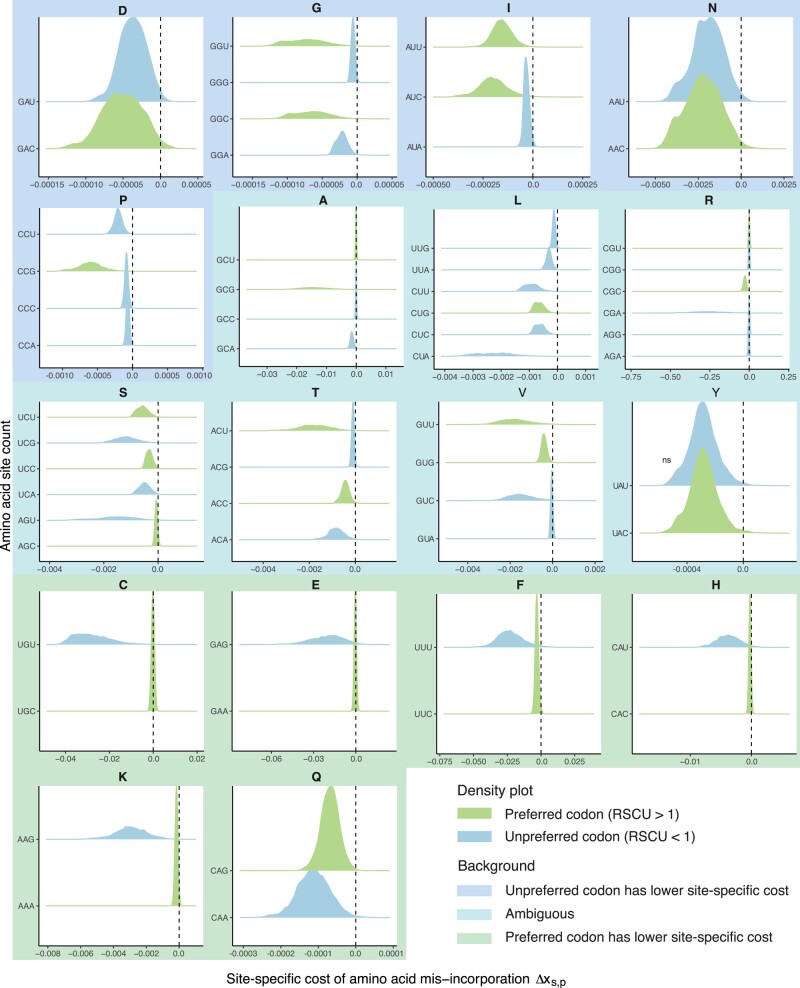
Distributions of site-specific costs of amino acid misincorporations differ between synonymous codons. Amino acids are grouped into three categories based on the differences in the distribution of site-specific cost of amino acid misincorporations of synonymous codons (indicated with a background color). Distribution color indicates if a codon is preferentially used (Relative Synonymous Codon Usage, RSCU > 1, green) or not (RSCU < 1, blue). While two codon amino acids always show a reduced impact of amino acid misincorporations for preferred codons (except for tyrosine), this is not true for other amino acids. In the cases of the three-codon amino acid isoleucine (I), the least preferred codon AUA shows the smallest impact of amino acid misincorporations. Similarly, a clear trend can be observed with the four-codon amino acid proline (P). In the case of valine (V), the impact of amino acid misincorporations does show a more varied pattern but coincides with the existence of the perfect matching tRNA. Note that the x-axes are shown at varying scales. *P*-values representing the comparison of the distributions indicate a significant difference for all distributions except for tyrosine in *E. coli* (supplementary table S4, Supplementary Material online).

In order to investigate the impact of synonymous codon usage on the fitness effect of error-prone translation, we compared the distribution of the site-specific cost of amino acid misincorporations $\Delta x_{s,p}$ for each group of synonymous codons separately (*E. coli* Fig. 4, *S. cerevisiae*  supplementary fig. S9, Supplementary Material online). We observed significant differences in the site-specific costs between synonymous codons. For example, in the *E. coli* genome, there is a large difference in the cost of amino acid misincorporations between sites with the phenylalanine codons UUU and UUC: Errors at UUC sites are less deleterious than at UUU sites ($p<1.0^{-16}$; Wilcox rank-sum test). Importantly, UUC is the preferred phenylalanine codon (as defined by Relative Synonymous Codon Usage, RSCU > 1). Similar effects can be observed for all other two-codon amino acids, with the preferred codon conferring generally less cost and therefore less severe fitness effects. The exception is tyrosine, which shows no significant difference between the synonymous codons (p=0.567; Wilcoxon’s rank sum test). In contrast, *S. cerevisiae* does not follow the same trend and often shows that the unpreferred codon (RSCU < 1) confers less cost (e.g. Cysteine; $p<1.0^{-16}$; Wilcoxon’s rank sum test). This may be an indication of a trade-off in codon usage of specific sites between translation fidelity and efficiency.

In either species, amino acids encoded by more than two codons do not show a clear trend in costs between preferred and unpreferred codons. For example, the sites using the unpreferred isoleucine codon AUA show the smallest costs, while the preferred AUC codon sites show the largest. Similar for glycine where both unpreferred codons (GGA, GGG) show smaller costs of amino acid misincorporations than the two preferred codons GGC and GGU in *E. coli*. This effect is even more exaggerated in *S. cerevisiae* with three unpreferred codons (supplementary fig. S9, Supplementary Material online). However, ordering between preferred and unpreferred codons might be less important when fitness effects between codons are small (Fig. 4, blue background; e.g. Aspartic acid (D)), but potentially matters greatly when fitness differences are large (Fig. 4, green background; e.g. Cysteine (C)). Amino acids with larger synonymous codon pools might be under more complex constrains (Fig. 4, cyan background). Overall, differences in costs and fitness effect between synonymous codons further suggest that local solutions, like adjusting codon usage, may be more common over global solutions to mitigate mistranslation events and their effects.

### Absence of tRNA Anticodons Reduces Amino Acid Misincorporations

So far, we considered the adaptation of the codon sequence to reduce amino acid misincorporations (local solution). We wanted to test if as an alternative, a global mechanism, the tRNA repertoire could also be adapted for higher fidelity. Interestingly, no organism harbors perfectly matching tRNA species for all codons (i.e. not all cognate anticodon–codon pairs exist). While this might serve as a mechanism to regulate translation speed (Ehrlich et al. 2021), it is not known if missing tRNA anticodons can reduce ambiguity during translation.

**Fig. 5.**
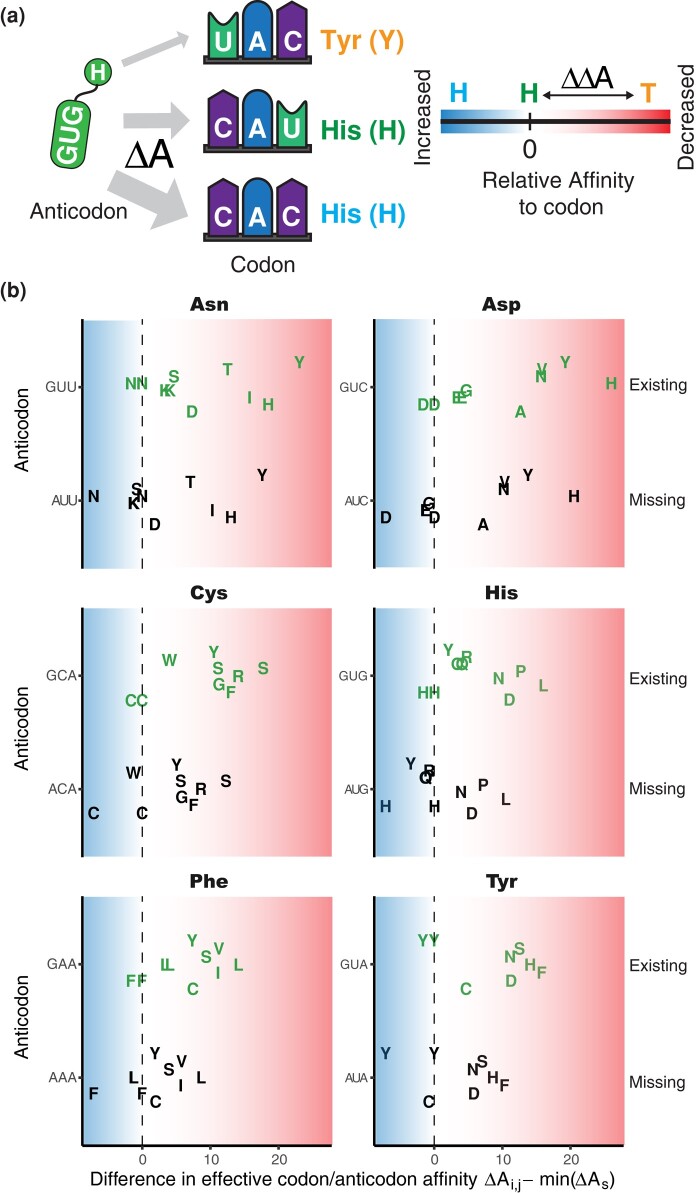
Minimizing tRNA misbinding by the available tRNA-anticodon pool. a) Schematics representing codon choice of the histidine tRNA with anticodon GUG. To minimize mistranslations, non-synonymous codons (UAC) should have a significantly lower effective binding affinity to the cognate anticodon than synonymous codons (CAU and CAC). Relative binding affinity is quantified based on the lowest synonymous effective binding affinity (min(ΔA_s_). Distance to 0 indicates the relative difference in binding affinity to the weakest synonymous codon/anticodon interaction. Negative values indicate stronger binding, positive values indicate weaker interactions. b) Impact of the tRNA pool on translation errors. Difference in effective codon/anticodon affinity (Δ*A_i, j_* is the effective binding affinity of codon *i* to anticodon *j*) for interactions with up to one codon/anticodon mismatch, for all two-codon amino acids with only one tRNA present in *E. coli*. The ribosome's ability to discriminate between correct and incorrect binding events is diminished if we assume that the tRNA with the *missing* anticodon (black) would be present instead of the tRNA with the *existing* anticodon (green) (see also supplementary fig. S9a, Supplementary Material online).

We tested if the effective binding affinity parameters differ between tRNA species present (existing tRNA anticodons) and absent (missing tRNA anticodons). Binding affinities for both existing and missing tRNA anticodons were calculated using Eq. (8), based on the model fit to the respective species. We investigated the difference in effective binding affinity of a given anticodon with its cognate (correct) and near-cognate (incorrect) codons (Fig. 5a). For example, the Asp-tRNA^GUC^ has a reduced binding affinity toward the near-cognate glutamine codons GAA and GAG relative to its cognate wobble codon GAU (74.4% and 68.9%, respectively). However, if we assume the Asp-tRNA would match its codon perfectly and carry the non-existent AUC anticodon instead, the affinity of the Asp-tRNA toward GAA and GAG increases to 114% and 113.3%, respectively, to the now wobble codon GAC (Fig. 5b). Similar effects can be observed for other amino acids encoded by exactly two codons in both *E. coli* and *S. cerevisiae* (Fig. 5b, supplementary fig. S10, Supplementary Material online). It is, therefore, possible that the presence/absence of tRNA anticodons in both organisms has been under selection to maximize the affinity difference between synonymous and non-synonymous codons and thus minimize amino acid misincorporation.

## Discussion

Detection and modeling of amino acid misincorporations at proteome scale enabled us to gain insights into the evolution of translation fidelity. Our mechanistic model of amino acid misincorporations, mTEL, was trained on misincorporations detected in existing mass spectrometry datasets (eTEL). We calculated error detection rates from these data and compared it to a comprehensive list of previous estimates from biochemical studies of individual codon positions (supplementary table S3, Supplementary Material online). While there are differences, overall our estimates match previous studies or predict lower error rates. For example, Kramer and Farabaugh (2007) estimated a misreading frequency for the Arginine codon AGG of $3.1\;\pm \;0.15\times 10^{-3}\;$ by Lys-tRNA^UUU^ at a single position in the firefly luciferase gene, that is very close to our estimated error detection rate for the same codon—by all tRNAs—of $6.27\;\pm \;3.84\times 10^{-3}$ (supplementary Data S2, Supplementary Material online). We compared our empirical error detection rate estimates of each codon (eTEL) to the previously obtained error rates from the only other high-throughput study (Mordret et al. 2019) and found no positive correlation (supplementary fig. S11a, Supplementary Material online, $R^{2}=0.04,\;\rho =0.141$, *E. coli*; supplementary fig. S11b, Supplementary Material online, $R^{2}=0.42,\;\rho =0.0002$, *S. cerevisiae*). Some of the discrepancies observed in our analysis and that of Mordret et al. may stem not only from the larger dataset of the current study, i.e. the observed amino acid substitutions (58,990 vs. 17,778), and the different data processing pipeline, but also from the definition of error rates. Mordret et al. estimated error rates by calculating the ratio of MS1 peak intensities of peptides with amino-acid misincorporations versus the peptides without. While comparing peak intensities works for peptides with similar properties, i.e. with conservative substitutions where amino acids have similar physicochemical properties, substitutions that strongly alter the physicochemical properties of an amino acid will change the relationship between peptide abundance and MS1 signal, preventing a direct comparison of peak intensities (Cech and Enke 2000). Additionally, comparing peak intensities with orders of magnitude differences may suffer from the dynamic range limitations of instruments (Arike et al. 2012) and low intensities introduce additional noise affecting quantification of protein abundance (Carrillo et al. 2010) (supplementary fig. S12a and b, Supplementary Material online). The error quantification method applied previously would overestimate error rates, sometimes as extreme as mistranslation being more likely than correct translation (by definition, these error estimates can be greater than one, e.g. AUA [isoleucine]) exhibiting an error rate greater than 1 (*E. coli*: supplementary fig. S11c, Supplementary Material online; *S. cerevisiae*: supplementary fig. S2a, Supplementary Material online, red bars). These estimates seem unrealistically high, meaning more mistranslation events than not (error rate >1). In contrast, here we calculated the error detection rate as the number of times codon *i* was observed to be mistranslated (aggregated over peptides) by the total number of times codon *i* was observed in any peptide. Our alternative estimate of error rates of codons using peptide count ratios is more stringent in detecting mistranslated peptides and based on orders of magnitude more observations from hundreds of datasets.

While mass spectrometry-based proteomics is the only method to detect amino acid misincorporations proteome-wide, it has limitations. Specifically, it is hindered by bias to detect only a fraction of the proteome (Nesvizhskii and Aebersold 2005), mainly high-abundant and “identifiable” peptides (Cech and Enke 2000; Fusaro et al. 2009), and the replicability and the concordance of experiments is low (Delmotte et al. 2009). In our work, the proteome coverage varied between 0.64% and 69.60% of all proteins in *E. coli* (1.49% to 76.41% in *S. cerevisiae*). Since most proteins are only identified via a single peptide, we cover only ∼7.59% of all *E. coli* codon positions (∼2.08% in *S. cerevisiae*), leaving a large number of positions unexplored. In addition, there is large variation in the number of times individual peptides are observed, ranging from 1 to 1,286 in *E. coli* (1 to 217 in *S. cerevisiae*).

We would like to note that the error rates of eTEL are influenced by selection on the protein. The peptides that we can observe are from proteins that have undergone selection already, e.g. degradation could eliminate some of the erroneous proteins that destabilize the protein or lead to aggregation. The genetic code itself is also already optimized for reducing deleterious misincorporations (Freeland and Hurst 1998). Therefore, the error rates we observe are not exactly the “mutation rates” of the translational machinery but rather the observed error rates after selection.

We overcome the limitations imposed by the low and biased coverage of mass spectrometry data by extrapolating error rates via our mechanistic model, mTEL. Nevertheless, our model has several simplifications. We focused on tRNA misincorporation as the main mechanism of amino acid misincorporation. However, amino acid misincorporations can be the result of tRNA mischarging, when the tRNA is charged by the wrong amino acid (Ling et al. 2009; Bilus et al. 2019). Since we cannot distinguish if a detected amino acid misincorporation is the result of mischarging or binding of incorrect tRNA, we assume that all observed amino acid misincorporations are the result of tRNA misbindings. Further, we approximated the tRNA concentrations in the cell using tRNA abundance estimates from RNA-seq data. However, at least in HEK cells, tRNA-seq revealed that tRNAs have different charging rates (Evans et al. 2017), thus the abundances of correctly charged tRNAs may differ from the RNA-seq-based estimates we used.

While our model has several simplifications and limitations, it emulates a complex mechanism to approximate the full landscape of amino acid misincorporations. This allowed us to perform *in silico* experiments not feasible in the laboratory. For example, we tested the impact of tRNAs with missing anticodons (Fig. 5b) and provide evidence that the absence of certain tRNAs in some species, such as Phe-tRNA^AAA^ or Asp-tRNA^AUC^, may serve to reduce amino acid misincorporations.

In agreement with previous observations in *E. coli* (Mordret et al. 2019; Sun and Zhang 2022), we found higher translational accuracies of more frequently used codons. Specifically, the relative synonymous codon usage (RSCU) is weakly negatively correlated with the relative error detection rate (*E. coli*: $R^{2}=0.27,\;\rho =0.0057,\;n=27$, supplementary fig. S13a, Supplementary Material online; *S. cerevisiae*: $R^{2}=0.16,\;\rho =0.042,\;n=27$, supplementary fig. S13b, Supplementary Material online).

We modeled the fitness landscape of proteins that includes the effects of amino acid misincoroprations as a product of the fitness effects of substitutions and their estimated frequency of occurrence (Eq. 3). We found that, while in *E. coli* translation errors are considered deleterious at 25.58% of assessed sites (Fig. 3d), translation errors in *S. cerevisiae* appear to impose a significant fitness burden at only about 5.75% of codon sites (supplementary fig. S8c, Supplementary Material online). When the fitness burden of mistranslation is conferred by a small number of deleterious sites and proteins, the preferred mechanism to cope with this fitness burden may be “local” adaptation of individual protein sequences, such as codon choice. It was previously proposed based on transcription error estimates that “local” solutions on a gene-by-gene basis can reduce the effects of errors (Rajon and Masel 2011). Here, based on data of proteome-wide translation errors, we detect codon choice as a “local” solution to amino acid misincorporations and describe how “global” solutions such as the presence or absence of tRNA species can increase translation fidelity.

While there are no experimental systems of error-free translation, hyper-accurate ribosomes are linked to lower growth rates (Ruusala et al. 1984). Assuming that translation fidelity is optimal, we can approximate the fitness burden of a hypothetical error-free translation system. To approximate the overall burden on the organism by considering its whole proteome, we sum over the fitness effects ($\Delta x_{p}$) of all proteins in the proteome. This results in an estimated organismal fitness effect of $\Delta x=\sum_{\forall p}\;\Delta x_{p}=-0.56$ for *E. coli* and Δx=−0.61 for *S. cerevisiae*, note that $\Delta x_{p}$ already accounts for relative protein abundance. Thus, both organisms face a similar estimated fitness effect of imperfect translation fidelity.

However, the efficacy of selection is dependent on the effective population size, which is vastly different between these organisms ($N_{e}=10^{8}$ in *E. coli* (Lynch 2010), $N_{e}=8,600,000$ in *S. cerevisiae* (Tsai et al. 2008)). To account for this, we computed the relative fixation probability of the error-prone translation system relative to a hypothetical error-free translation system (see Methods) that resulted in $\Theta (\Delta x)=2\times 10^{-19}\;$ for *E. coli* and Θ(Δx)=0.0561 for *S. cerevisiae*. Thus, *E. coli* has a greater potential to overcome this fitness burden, as its disadvantage to a hypothetical competitor with perfect translation fidelity is much greater. This appears to be in line with expectations derived from the nearly neutral theory (Ohta 1996; Ohta and Gillespie 1996), where the range of *s* of substitutions to be considered neutral declines with the increase in effective population size as the effective population size of *E. coli* is orders of magnitude larger than that of *S. cerevisiae*.

The error-aware fitness landscape presented here will facilitate our understanding of how misincorporations, also called phenotypic mutations, contribute to molecular evolution. It was proposed that phenotypic mutations can serve as “look ahead mutations” when multiple mutations are required for a novel trait (Whitehead et al. 2008). The advantageous misincorporations may allow otherwise deleterious intermediates to fixate by “flattening” the fitness landscape. Our model can be used to design mutations to test the above hypotheses.

Our models are applicable to any organism. There is a large variability in translation fidelity across organisms as measured by individual reporter assays (Romero Romero et al. 2022). Recent advances, including this study, make it now possible to explore translation accuracy proteome-wide in many species, as well as study translation accuracy under changing conditions, such as stress (Mohler and Ibba 2017) or diseases (Kirchner and Ignatova 2015; Goodarzi et al. 2016; Pataskar et al. 2022), and assess their evolutionary impact.

## Materials and Methods

### Detecting Amino Acid Misincorporations via Open Search

In total, 146 *E. coli* and 185 *S. cerevisiae* datasets were downloaded from PRIDE (Jones and Côté 2008; Perez-Riverol et al. 2018, 2022) (last accessed: 16.11.2022). Note, we only downloaded datasets tagged with a single species and are not marked as containing isotopic labeling (e.g. SILAC experiments). First, they were analyzed in bulk by using the dry-run functionality of the Fragpipe (version 17) software suite to extract the commands executed by Fragpipe. The individual tools included in Fragpipe were then called from a bash script on a computing cluster, searching each PRIDE project in parallel. Options are based on the settings used in the predefined “Open Search” of Fragpipe. All raw files (Thermo .raw format) in a dataset were then searched together by MSFragger (Kong et al. 2017) (version 3.4) open search (Yu et al. 2020) against the *S. cerevisiae* proteome (translated reference CDS from yeastgenome.org, last accessed 12.03.2020) with mitochondrial genes removed, and the *E. coli* proteome (extracted from genome and translated; genome obtained from NCBI, last accessed 15.09.2020). MSFragger's peptide identifications were then post-processed and filtered by Crystal-C (Chang et al. 2020), PeptideProphet, ProteinProphet, and Philosopher (da Veiga Leprevost et al. 2020) (see supplementary methods for details).

### Detecting Substitutions in Open Search Results

Amino acid substitutions were detected with a custom Python script (version 3.9). For each dataset, False Discovery Rate (FDR)-filtered psm.tsv files generated by Philosopher were collected. Peptides were further filtered to remove those that were matched to several proteins. Peptides with a mass shift between −5 and +5 mDa were considered as unmodified, and all others as modified. Modified peptides were only retained if the modified position in the protein sequence was covered by an unmodified peptide present in the same MSFragger file (identified in the same MS measurement) and if the position of the modification could be unambiguously localized. All remaining peptides with a mass shift matching the mass difference between the original and a different amino acid were marked as substitutions, and the original and substituted amino acid were annotated, with leucine and isoleucine treated as equivalent. All peptides with a mass shift and localization also matching a known PTM (see Supplementary material for list of PTMs) were removed.

### Curation of Mass Spectrometry Datasets

We removed datasets with less than 10 detected amino acid substitutions and an enrichment (>10% of detected amino acid misincorporations) of mass differences indicative of isotopic labeling. Remaining datasets were manually curated (supplementary Data S1, Supplementary Material online) and datasets with potential biases in translation fidelity, e.g. due to antibiotic treatment (Wohlgemuth et al. 2021) or mutated tRNA (Bilus et al. 2019), were removed. The final dataset size was 80 for *E. coli* and 72 for *S. cerevisiae*.

### Calculating Error Detection Rates

For each codon, an error detection rate was calculated as the total number of times (number of Peptide Spectrum Match [PSM]) peptides covering a position with this codon and carrying a substitution at that position were detected divided by the total number of times peptides, modified or unmodified, covering a position with this codon were detected.

### Exploring the Relationship Between Codon Usage and Translation Fidelity

Relative synonymous codon usage (RSCU) was calculated from the top 5% expressed using integrated data from PAXdb (Wang et al. 2015) for *E. coli* and *S. cerevisiae* genes using the function uco in the R (4.2) package seqinr (4.2 to 23).

### Mechanistic Translation Error Model

mTEL is designed to predict tRNA misincorporation rates at steady state. mTEL is parameterized using tRNA abundance, 16 (20 for *E. coli* as the lysidine nucleotide in the tRNA is considered) effective nucleotide affinity parameters, and two site-specific important parameters (Fig. 2b). mTEL considers tRNA competition for arrival at the ribosome determined, the binding of the tRNA and the repeated arrival of competing tRNAs. The probability of tRNA incorporation is calculated then based on the individual arrival and binding probabilities as described in detail below.

### tRNA Arrival Probabilities

While we assume the binding probabilities of the tRNAs at stationarity, we have to consider the arrival rates of each tRNA at the ribosome. We assume that the waiting time for a tRNA to arrive at the ribosome is exponentially distributed with the rate being proportional to the tRNA abundance. tRNA abundance was obtained from Weinberg et al. (2016) and Larson et al. (2014) for *S. cerevisiae* and *E. coli*, respectively. The rate parameter for the exponential distribution is calculated following Fluitt et al. (2007). Briefly, we assume that a cell can be discretized into $n=V/l^{3}$ locations where *V* is the cell volume (e.g. $0.6\times 10^{-18}m^{3}$ for *E. coli* and $4.2\times 10^{-17}m^{3}$ for *S. cerevisiae*) and *l* is the effective length of a tRNA ($1.58\;\times \;10^{-8}m$) (Weinberg et al. 2016). Assuming a transition time of $\tau =l^{2}/6*D$, where *D* is the diffusion coefficient for tRNA ($8.42\times 10^{-11}m^{2}$) (Weinberg et al. 2016), the arrival rate can be expressed as


(5) $$\lambda_{i}=\frac{P_{i}}{\tau }$$

where $P_{i}=[tRNA_{i}]/n$ is the probability of tRNA *i* to occupy a given position in the cell. Given the rate at which a tRNA arrives at the ribosome, we can calculate the probability that tRNA_i_ will arrive before tRNA_j_ as


(6) $$P_{i<j}=\frac{\lambda_{i}}{\lambda_{i}+\lambda_{j}}$$

(see arrival probabilities for all existing tRNAs in *E. coli* in supplementary fig. S6a, Supplementary Material online). Equation (6) also holds when comparing the arrival rate $\lambda_{i}$ to any set of competitors by simply adding their rates *λ* to the denominator. For example, the probability to arrive first is


(7) $$P_{\;first}^{i}=\frac{\lambda_{i}}{\sum_{\forall k}\;\lambda_{k}}$$

and the probability to not arrive first is $P_{\bar{\;first}}^{i}=1-P_{\;first}^{i}$.

We can also calculate the number of arrival events for tRNA *i* we expect to occur before another tRNA *j* to arrive. Since tRNA *i* is distributed with rate $\lambda_{i}$ and tRNA j is distributed with rate $\lambda_{j}$ the expected number of arrival events is $\frac{1}{\lambda_{i}}$ and $\frac{1}{\lambda_{j}}$, respectively. Therefore, the expected number of arrival events of tRNA *i* before *j* is $\frac{\lambda_{i}}{\lambda_{j}}$.

### tRNA Binding Probabilities

The binding probability of anticodon *j* to codon *i* depends on the effective binding affinity $\Delta A_{i,j}$. Binding is parameterized based on the individual nucleotide effective binding affinities $a_{c_{k}^{i},ac_{k}^{j}}$, where $a_{c_{k}^{i},ac_{k}^{j}}$ is the effective binding affinity between the nucleotide *c* of codon *i* at position *k* and the anticodon *j* nucleotide *ac* at position *k*. Effective binding affinities are further scaled by site-specific importance terms $s_{k}$, describing how much weight should be given to a site, relative to the wobble position ($s_{3}=1$). Thus, the effective binding affinity for each codon/anticodon pair i,j can be calculated as


(8) $$\Delta A_{i,\;j}=\overset{3}{\underset{k=1}{\sum }}\;a_{c_{k}^{i},\;ac_{k}^{j}}s_{k}$$

The binding probability of tRNA *j* binding at codon *i* at equilibrium is then calculated as the partition function, which determines the probability of a state given its free energy (affinity),


(9) $$P_{b}^{i,\;j}=\frac{\exp[-\Delta A_{i,\;j}]}{\sum_{\forall k}\;\exp[-\Delta A_{i,\;k}]}$$

where the denominator is the sum over the binding affinities for all tRNAs present in the cell.

### tRNA Incorporation Probability

To calculate the incorporation probability $P_{I}^{i}$ of a tRNA *i*, we have to distinguish two cases. First, the tRNA *i* in question arrived before any competitor. Second, any competing tRNA *k* arrived before. In the first case, the probability to incorporate tRNA *i* is simply the probability to bind, $P_{I|first}^{i}=P_{b}^{i}$. In the second case, however, we have to consider that tRNA *k* is expected to have arrived before the focal tRNA *i*. Therefore, *i* being incorporated despite *k* arriving beforehand requires that *k* does not bind $\left(1-P_{b}^{k}\right)$. However, depending on relative arrival rates, *k* may arrive multiple times before *i*. Assuming that arrival event of tRNAs is independent and arrival times are exponentially distributed with arrival rate λ then we expect that tRNA *k* will arrive $\lambda_{k}/\lambda_{i}$ times before the focal tRNA *i*. Since we assume independent binding probabilities, the probability that *k* does not bind $\lambda_{k}/\lambda_{i}$ times is then $(1-P_{b}^{k}{)}^{\frac{\lambda_{k}}{\lambda_{i}}}$. Considering all possible competitors, tRNA *i* being incorporated when not arriving first, requires the focal tRNA *i* to bind ($P_{b}^{i}$), and all other tRNA *k* arriving beforehand to not bind:


(10) $$P_{I|\bar{\;first}}^{i}=P_{b}^{i}\times \underset{\forall k}{\prod }\;(1-P_{b}^{k}{)}^{\frac{\lambda_{k}}{\lambda_{i}}}$$

Finally, we add the two cases (tRNA *i* incorporating given that *i* is arriving first and tRNA *i* incorporating given that *i* is not arriving first) using the law of total probability:


(11) $$P_{I}^{i}=P_{I|first}^{i}\times P_{\;first}^{i}+P_{I|\bar{\;first}}^{i}\times P_{\bar{\;first}}^{i}$$

### Model Fitting

We fitted the mTEL model using Markov Chain Monte Carlo with Gibbs sampling and unbound uniform priors on the linear scale on all parameters. The Markov chain was estimated for 10,000 steps after an initial burn-in period of 5,000 steps. Only every 10th step was retained and the last 500 thinned samples were used as posterior distribution.

### Calculate Error-free Translation Probability

We calculated the incorporation probability ($p_{i}$) of each tRNA to each codon based on available tRNA abundance data estimated from RNAseq data for *E. coli* and *S. cerevisiae*, respectively (Weinberg et al. 2016; Wei et al. 2017), and based on our estimates of codon/anticodon effective binding affinities. The probability of incorporating a synonymous tRNA (cognate tRNAs that carry the correct amino acid) at a given position can then be calculated as


(12) $$P[tRNA_{s}]=1-\underset{\forall tRNA_{n}}{\sum }\;P_{i}$$

where $tRNA_{s}$ is the set of synonymous tRNAs (and $tRNA_{n}$ is the set of non-synonymous tRNAs (non-cognate tRNAs). As we are assuming site independence, the probability of translating a protein error free ($P_{f}$) is then simply


(13) $$P_{f}=\overset{L}{\underset{i=1}{\prod }}\;1-P_{i}[tRNA_{N}]$$

where *L* is the length of protein in question.

### Calculating Fitness Effects

Fitness estimates of amino acid substitutions were calculated with EVmutations (Hopf et al. 2017), as implemented in the EVcouplings python package (version 0.0.5) (Hopf et al. 2018). The fitness estimates of EVmutation are likely closer to organismal fitness than molecular fitness since they are based on multiple sequence alignments from diverse organisms. We collected all Uniprot IDs assigned to the *S. cerevisiae* and *E. coli* reference proteomes. The EVcouplings alignment step was performed for each protein with bit scores 0.1, 0.2, 0.3, 0.4, 0.5, and the best alignment was chosen following the process in Luppino et al. (2023) (see Supplementary Methods). Fitness effects of all amino acid substitutions were estimated as ΔE(σ) using EVmutation (Hopf et al. 2017) based on co-evolution and conservation of residues. Site-specific fitness effects $\Delta x_{s,p}$ of translation errors were then defined as the average amino acid fitness at a given position, weighted by the misincorporation probability for that amino acid. The evolutionary effect of each amino acid misincorporation was assessed following the fixation probability definition of Sella and Hirsh (Sella and Hirsh 2005)


(14) $$\Theta_{f}=\frac{2S}{1-exp(-2S)}$$

where $S=q\times N_{e}\times \Delta x$. The effective population size was assumed to be $N_{e}=8,600,000$ in *S. cerevisiae* (Tsai et al. 2008) and $N_{e}=10^{8}$ in *E. coli* (Lynch 2010). The scaling parameter $q=4.19\times 10^{-7}$ is a constant, scaling the cost of protein production (Gilchrist 2007; Gilchrist et al. 2015). Protein-specific fitness effects ($\Delta x_{p}$) were calculated as the sum of the site-specific fitness effects ($\Delta x_{s,p}$) weighted by the relative contribution of a protein to the proteome based on its abundance. Relative protein abundance $\phi_{p}$ of a protein was derived from the integrated data from PAXdb (Wang et al. 2015) for *S. cerevisiae* and *E. coli*. Protein specific evolutionary effects were assessed in the same way as site-specific effects.

**Statistical analysis and data visualization** was performed with R. For all box plot representations, thick black line indicates median, box indicates 25th and 75th percentiles, and whiskers indicate 1.5 times the inter-quartile range.

## Supplementary Material

Supplementary material is available at *Molecular Biology and Evolution* online.

## Supplementary Material

msae048_Supplementary_Data

## Data Availability

The mTEL pipeline and the mTEL model developed and used as part of this manuscript are available at https://git.mpi-cbg.de/tothpetroczylab/deTEL. We also provide a singularity container and all the Datasets (1 to 54) at https://doi.org/10.17617/3.6RVSR5.
