# Supplementary material for: Fitness Effects of Phenotypic Mutations at Proteome-Scale Reveal Optimality of Translation Machinery
Source: Mol Biol Evol. 2024 Feb 29;41(3):msae048. doi: 10.1093/molbev/msae048 (PMC10939442; doi:10.1093/molbev/msae048)
Supplement: msae048_Supplementary_Data [file msae048_supplementary_data.zip › Landerer_MBE_supplementary_material_accepted.pdf]

Supplementary Information

Fitness effects of phenotypic mutations at proteome-scale reveal optimality of translation machinery

Cedric Landerer[1,2], Jonas Pöhls[1,2], Agnes Toth-Petroczy[1,2,3,*]

Affiliations:
[1]Max Planck Institute of Molecular Cell Biology and Genetics, 01307 Dresden, Germany
[2]Center for Systems Biology Dresden, 01307 Dresden, Germany
[3]Cluster of Excellence Physics of Life, TU Dresden, 01062 Dresden, Germany
[*]Corresponding author: toth-petroczy@mpi-cbg.de

Content:
Supplementary Methods
Supplementary Figures 1-13
Supplementary Data 1-4 and Tables 1-4
Supplementary References

Supplementary Methods

Detecting amino acid misincorporations via open search

Datasets downloaded from PRIDE (Jones and Côté 2008; Perez-Riverol et al. 2018; Perez-Riverol et al. 2022) (last accessed: 04.11.2021) were analyzed in bulk by first using the dry-run functionality of the Fragpipe (version 17) software suite to extract the commands executed by Fragpipe. The individual tools included in Fragpipe were then called from a bash script on a computing cluster, searching each PRIDE project in parallel. The configuration files and command line options are based on the settings used in the predefined 'Open Search' workflow in the GUI version of Fragpipe. For each dataset, a 'workspace' was first initialized by Philosopher. All raw files (Thermo .raw format) in the dataset were then searched together by MSFragger (Kong et al. 2017) (version 3.4) open search (Yu et al. 2020) against the *S. cerevisiae* proteome (translated reference CDS from yeastgenome.org, last accessed 12.03.2020) with mitochondrial genes removed and the *E. coli* proteome (extracted from genome and translated, genome obtained from NCBI, last accessed 15.09.2020).

MSFragger was set to perform mass calibration, deisotoping and to remove neutral losses. The precursor tolerance was 5 ppm, MS/MS tolerance was 10 ppm. The range of mass shifts (precursor mass window) was set to -135 to 135 Da, excluding the range from -1.5 to +3.5 Da. MSFragger was set to allow for any mass shift that fell within this range and was assigned to a amino acid. In-silico digestion was specified as 'strict trypsin' (cleave after K and R, regardless of C-terminal amino acid), with up to two missed cleavages. Only fully tryptic peptides with a length of 7 – 50 amino acids, a mass of 500 – 5000 Da, and a precursor charge of +1 – 4 were allowed. A fixed modification of Cysteine with mass 57.02146 (carbamidomethylation), and no variable modifications. All other MSFragger settings were kept default. MSFragger output (.pepXML and .tsv files) were move to a dataset-specific folder and processed by CrystalC (Chang et al. 2020) (version 1.3.2) to identify missed cleavages, semi-enzymatic peptides and chimeric spectra among the MSFragger identifications. Precursor charge was set to +1 – 6, precursor mass tolerance to 20 ppm, and the precursor isolation window to 0.7 Da. The number of theoretical isotope peaks was set to 3, and isotope error correction was disabled.

PeptideProphet was then used to score the peptides retained by CrystalC. The c.pepXML files produced by CrystalC were analysed with the options —nonparam, --decoy, --decoyprobs and –expectscore enabled, the mass width set to 1000 and the clevel set to 2. PeptideProphet(da Veiga Leprevost et al. 2020) output was combined into a single .interact.pepXML file. ProteinProphet (da Veiga Leprevost et al. 2020) was then executed on PeptideProphet’s output to score proteins, with the maximum ppm difference set to 2,000,000 (standard in the Open Search) and combining the output. The Philosopher (da Veiga Leprevost et al. 2020) commands ‘annotate’, ‘filter’, and ‘report’ were called to produce additional reports. ‘annotate’ was called on the sequence database with standard parameters. ‘filter’ was called on the .pepXML and .protXML files generated by PeptideProphet and ProteinProphet, respectively, with the –sequential, --razor, and –mapmods options enabled, and the protein-level FDR set to 0.01. ‘report’ was called with the —decoys option enabled. The workspace was cleaned up by the ‘workspace’ command. A summary of the identified mass shifts was generated with PTM-Shepherd (Geiszler et al. 2021) (version 1.0). All options were left at default, the only specified variable modification was failed carbamidomethylation with a mass of -57.021464.

Detecting substitutions in open search results

Amino acids substitutions were detected with a custom Python script (version 3.9) (https://git.mpi-cbg.de/tothpetroczylab/detel). All generated and FDR filtered psm.tsv files were collected. Peptides that matched to several proteins were removed. All peptides were annotated with their start and end position in the corresponding protein. Peptides with a mass shift between -5 and +5 mDa were considered as unmodified, and all others as modified. PSMs associated with unmodified peptides were retained and all remaining decoys were removed.

Modified peptides were only retained if the unmodified peptide was present in the same MSFragger file (identified in the same MS measurement) and if the position of the modification could be unambiguously localized. Remaining peptides with a mass shift matching the mass difference between two amino acids were marked as substitutions, and the original and substituted amino acids were annotated, with Leucine and Isoleucine treated as equivalent (due to identical mass). Additionally, all peptide with a mass shift and localization also matching a known PTM were removed.

tRNA arrival probabilities

While we assumed the binding probabilities of the tRNAs at stationarity, we had to consider the arrival rates of each tRNA at the ribosome. We assumed that the waiting time for a tRNA to arrive at the ribosome is exponentially distributed with the rate being proportional to the tRNA abundance. tRNA abundance was obtained from Weinberg et al (Weinberg et al. 2016) and Larson et al(Larson et al. 2014) for *S. cerevisiae* and *E. coli*, respectively. The rate parameter for the exponential distribution is calculated following (Fluitt et al. 2007). Briefly, we assume that a cell can be discretized into $n = V/l^3$ locations where V is the cell volume (e.g. $0.6 \times 10^{-18} m^3$ for *E. coli* and $4.2 \times 10^{-17} m^3$ for *S. cerevisiae*) and l is the effective length of a tRNA ($1.58 \times 10^{-8} m$) (Weinberg et al. 2016). Assuming a transition time of $\tau = l^2/6 * D$, where D is the diffusion coefficient for tRNA ($8.42 \times 10^{-11} m^2$)(Weinberg et al. 2016) the arrival probability can be expressed as:

$$\lambda_i = \frac{P_i}{\tau}$$

were $P_i = [tRNA_i]/n$ is the probability of tRNA i to occupy a given position in the cell.

Given the rate at which a tRNA arrives at the ribosome, we can calculate the probability that tRNA a will arrive before tRNA b. We consider the joint probability of two tRNAs arriving at the ribosome.

$$f_{a,b} = f_{A|B}(a|b)_{*f_B(b)}$$

$$\int_a \int_b f_{a,b} db\, da = 1$$

we can calculate the probability of a arriving before b as

$$p_{a<b} = \int_{a=0}^{\infty} \int_{b=a}^{\infty} f_{a,b} db\, da$$

$$= \int_{a=0}^{\infty} f_A(a) F_B(a) da$$

$$= \int_{a=0}^{\infty} \mu e^{-\mu a}\left(1 - e^{-\lambda a}\right) da$$

$$= \frac{\lambda}{\lambda + \mu}$$

Similarly, for many competitors, it holds that

$$\int_{a} \cdots \int_{z} f_{a,\cdots,z} dz \cdots da = 1$$

and we can express the probability of a focal tRNA a to arrive before any other tRNA as

$$p_{a,\text{first}} = \frac{\lambda_a}{\sum_i \lambda_i}$$

Calculating fitness effects

EVcouplings (version 0.0.5) was used to compute fitness estimates of amino acid substitutions. We collected all Uniprot IDs assigned to the *S. cerevisiae* and *E. coli* reference proteomes. The EVcouplings (Hopf et al. 2018) alignment step was performed for each protein with bit scores 0.1, 0.2, 0.3, 0.4, 0.5. The alignment with the best performing bit score was selected for the fitness estimation. Specifically, we calculated the the skewness of the Evolutionary Couplings (EC) distribution obtained at each bitscore threshold, and selected the one with the highest skewness of the EC distribution following (Luppino et al. 2023). Fitness effects of all amino acid substitutions were estimated as $\Delta E(\sigma)$ using EVmutation (Hopf et al. 2017) based on co-evolution and conservation of residues. Site specific fitness effects $\Delta x_{s,p}$ of translation errors were then defined as the weighted average of the fitness effects of all 20 amino acids at a given position

$$\Delta x_{s,p} = \sum_{\forall a \in AA} p_a \, \Delta E\left(\sigma_a^{s,p}\right)$$

were p_a is the probability of the amino acid misincorporation. The evolutionary effect of each amino acid misincorporation was assessed following the fixation probability definition of Sella and Hirsh (Sella and Hirsh 2005). The relative fixation probability of the observed fitness effects Δx to a hypothetical error free translation system serving as a null hypothesis evaluated as

$$\Theta(\Delta x) = \frac{\left(1 - \exp(-2\Delta x q)\right)N_e}{1 - \exp(-2N_e \Delta x q)}$$

where the effective population size is $N_e = 8{,}600{,}000$ in *S. cerevisiae* (Tsai et al. 2008) and $N_e = 10^8$ in *E. coli* (Lynch 2010), $q = 4.19 \times 10^{-7}$ is a constant, scaling the cost of protein prodution (Gilchrist 2007).

Protein specific fitness effects Δx_pwere calculated as the sum of the site-specific fitness effects $\Delta x_{s,p}$ weighted by the relative contribution of a protein to the proteome based on its abundance.

$$\Delta x_p = \sum_{\forall s} \phi_p \, \Delta x_{s,p}$$

The relative protein abundance ϕ_p of a protein was derived from the integrated data from PAXdb (Wang et al. 2015) for *S. cerevisiae* and *E. coli*, respectively. Protein specific evolutionary effects were assessed in the same way as site-specific effects.

Supplementary Figures

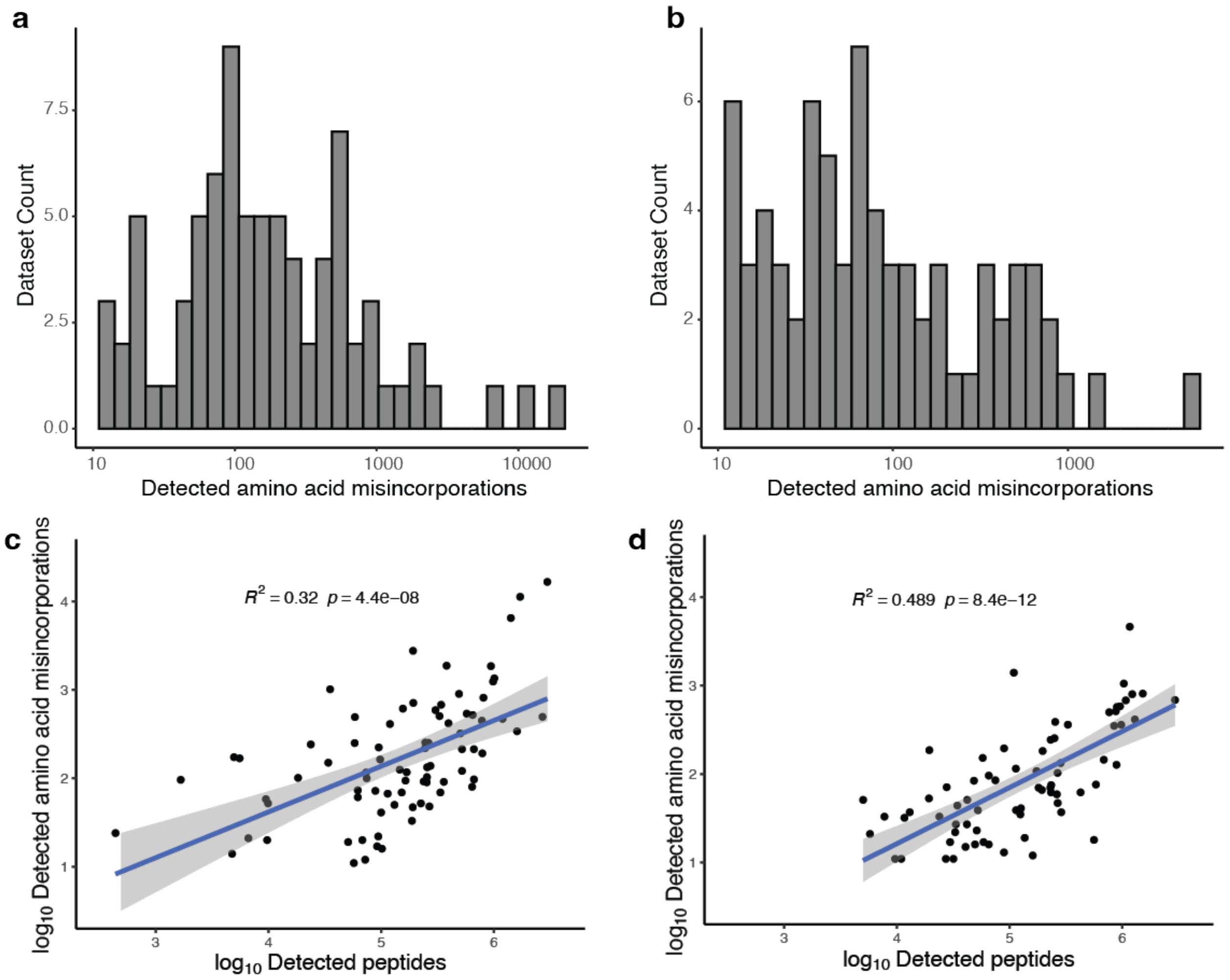

Figure S1: Amino acid misincorporations are abundant in many existing mass-spectrometry datasets. a, b) Histogram of the number of identified amino acid misincorporations in **a)** *E. coli* (N = 80) and **b)** *S. cerevisiae* (N = 72) datasets (Data S1). **c, d)** Comparison of identified amino acid misincorporations with the number of total peptides detected in the filtered **c)** *E. coli* **d)** *S. cerevisiae* datasets (Data S1).

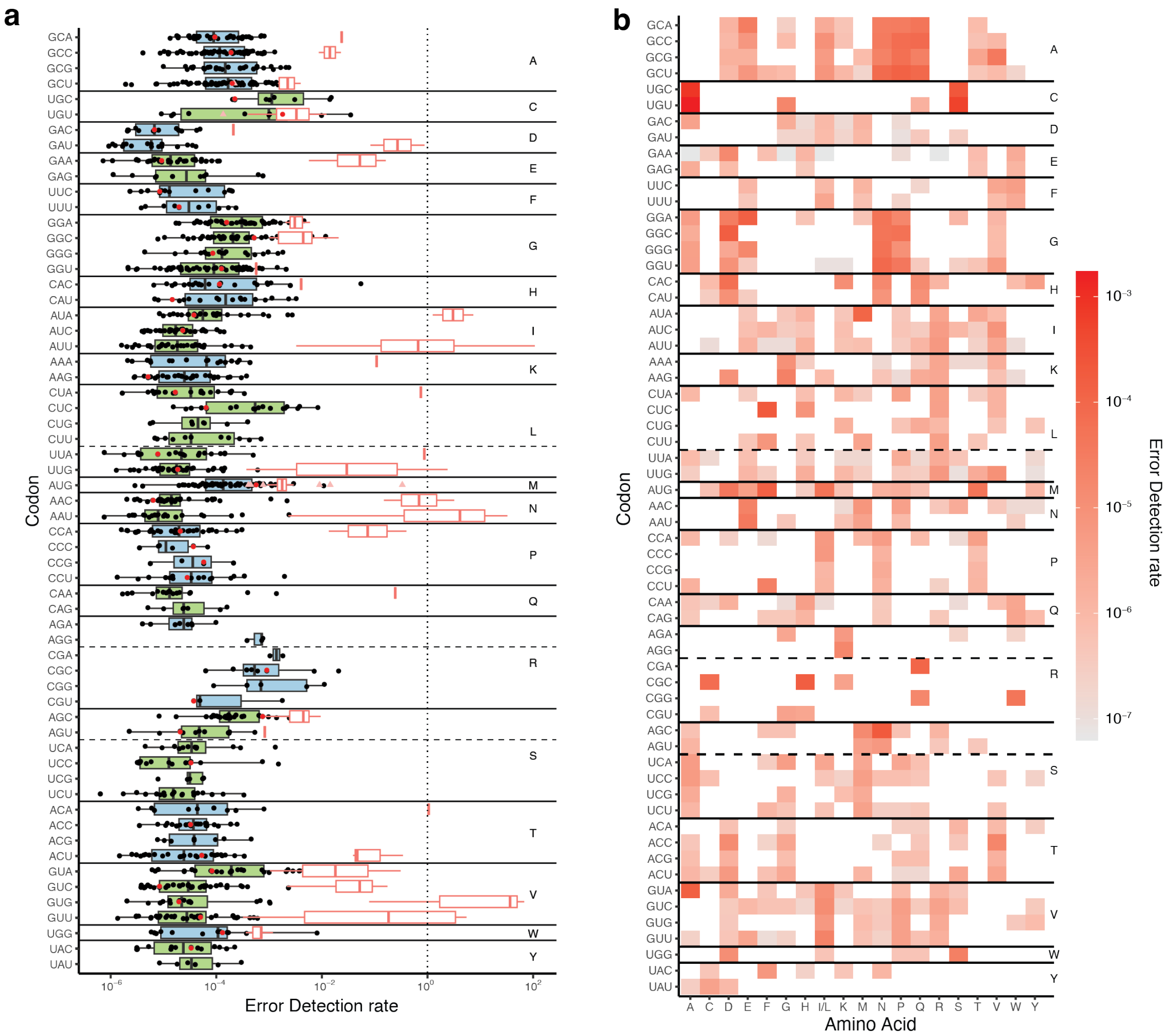

Figure S2: Amino acid misincorporation rates vary between codons and datasets. a) Codon specific error detection rates in *S. cerevisiae* datasets (thick black line indicates median, box indicates 25th and 75th percentiles, whiskers indicate 1.5 times the inter-quartile range). For reference, red dot indicates our detection rate for the *S. cerevisiae* data used by (Mordret et al. 2019). Red bar graph shows error rates estimated by Mordret et al. (2019). **B)** Heatmap of all codon specific amino acid misincorporation rates pooled across all filtered *S. cerevisiae* datasets (Data S2). Leucine and Isoleucine cannot be distinguished due to their identical mass. Each cell shows the rate of amino acid i misincorporated at codon j. White indicates no observation.

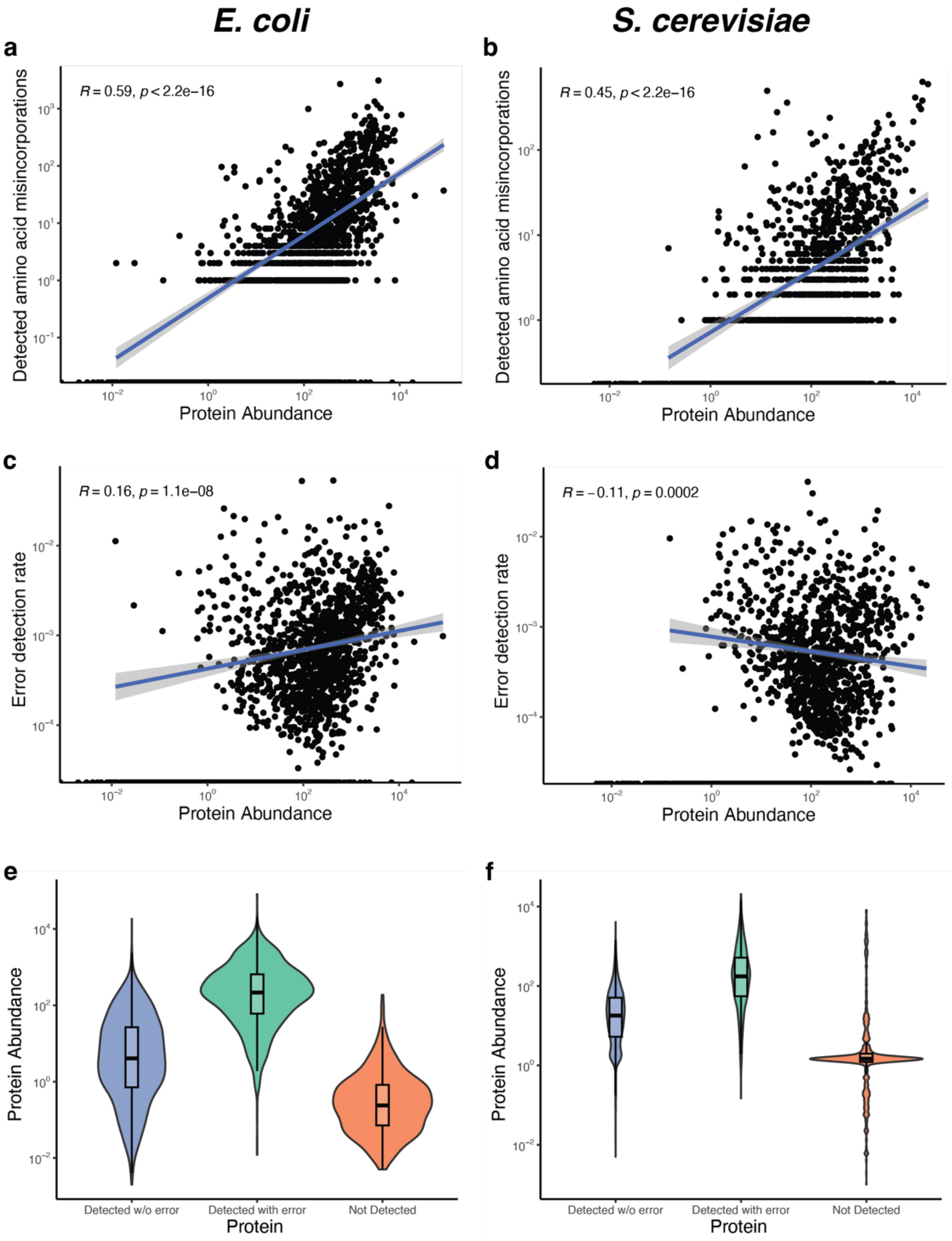

Figure S3: Amino acid misincorporation are more likely to be detected in highly abundant proteins. a, b) The number of detected amino acid misincorporations increase with protein abundance based on PaxDB (Wang et al. 2015), in both **a)** *E. coli* and **b)** *S. cerevisiae*. **c, d)** Amino acid misincorporation rates show a weaker correlation with protein abundance than the number of amino acid misincorporation in both **c)** *E. coli* and even negative correlation in **d)** *S. cerevisiae*. This indicates that highly abundance proteins do not experience higher error rates. **e, f)** More abundant proteins are more likely to be detected in mass spectrometry in general (higher coverage), and therefore they are more likely to be detected harboring an amino acid misincorporation both in **e)** *E. coli* and **f)** *S. cerevisiae*.

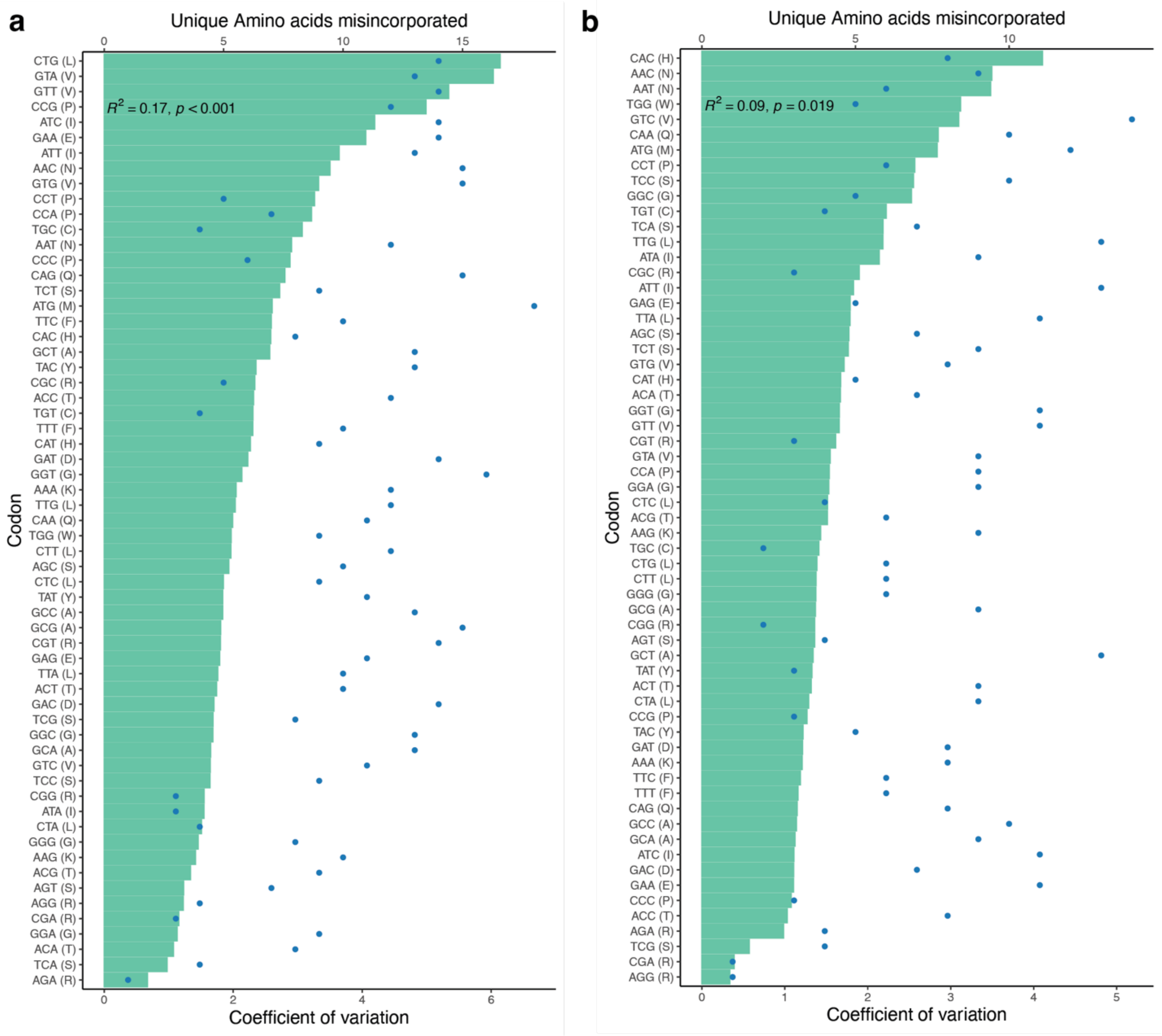

Figure S4: Large difference in variation between codon. a, b) Coefficient of variation (green bars) of the detection rate of a codon and the number of unique amino acids misincorporated (blue dots) at that codon for **a)** *E. coli* and **b)** *S. cerevisiae*. Pearson correlation and significance level between number of unique amino acids misincorporated and Coefficient of variation is indicated.

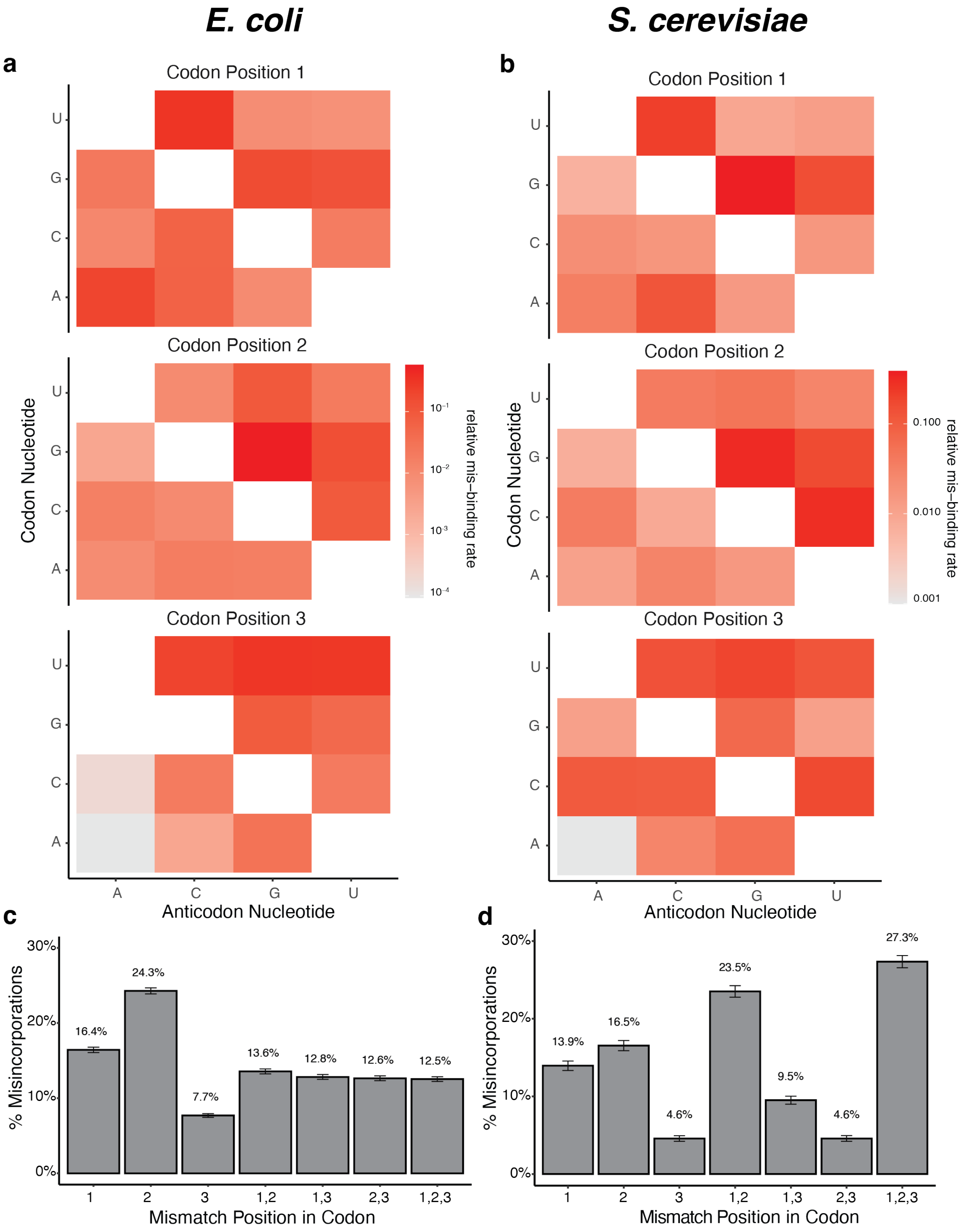

Figure S5: Amino acid misincorporations are caused by all types of nucleotide mismatches. **a, b)** Nucleotide mis-binding rate at each codon position for **a)** *E. coli* and **b)** *S. cerevisiae*. **c, d)** Percentage of observed amino acid misincorporation caused by a given codon position in **c)** *E. coli* and **d)** *S. cerevisiae*. Most single nucleotide mismatches are observed at the second codon position. Error bars show 99 percentiles obtained by bootstrapping observed misincorporations.

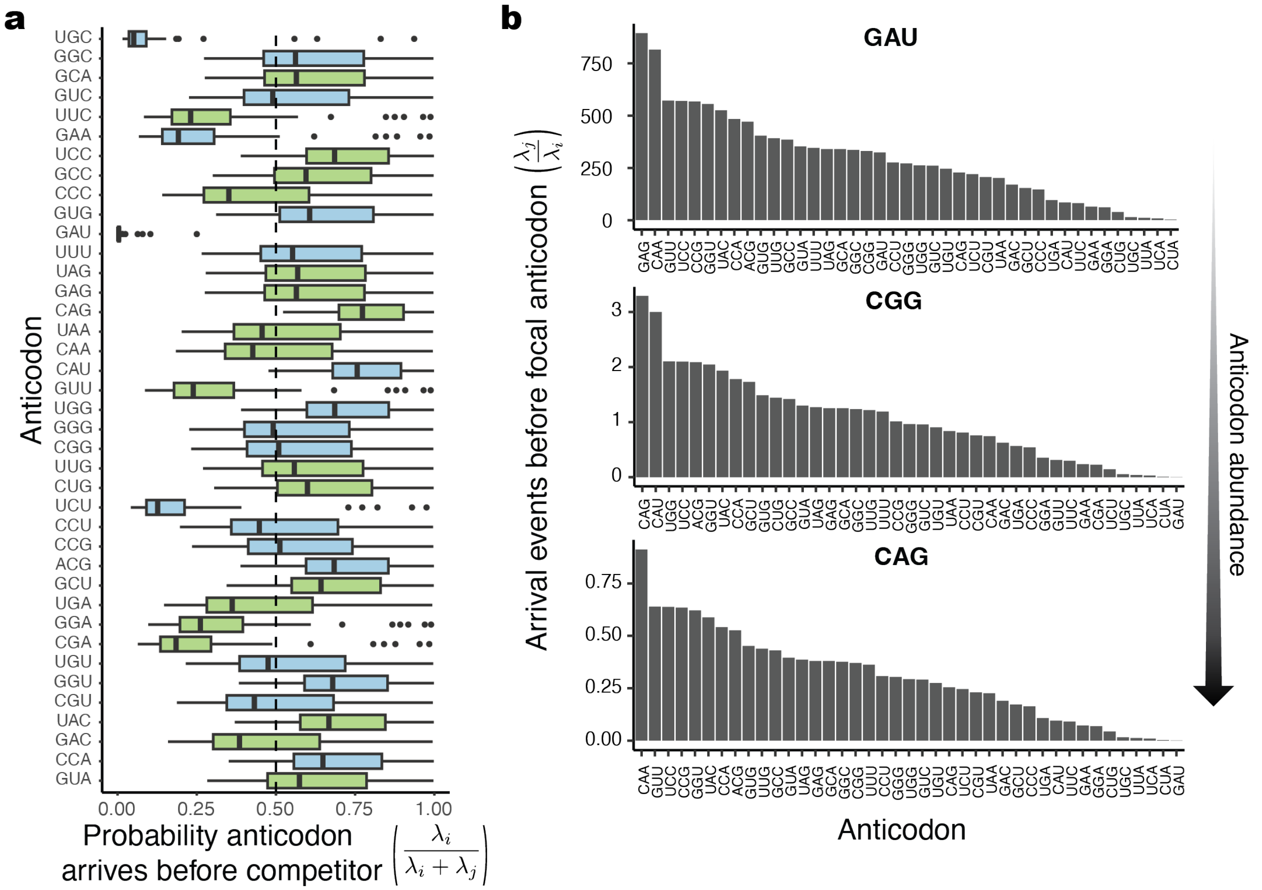

Figure S6: Abundance determines differences in arrival probabilities and events between competing tRNAs. **a)** Distribution of probabilities of a focal tRNA I to arrive before any potential competitor. Low abundance tRNA like UGC and GAU have low probabilities to arrive before other tRNA. In contrast, high abundance tRNA such as CAG are likely to arrive before any other tRNA. **b)** Expected number of competing tRNA arrival events before the focal tRNA arrives for three examples of focal codons with low, medium, and high abundance.

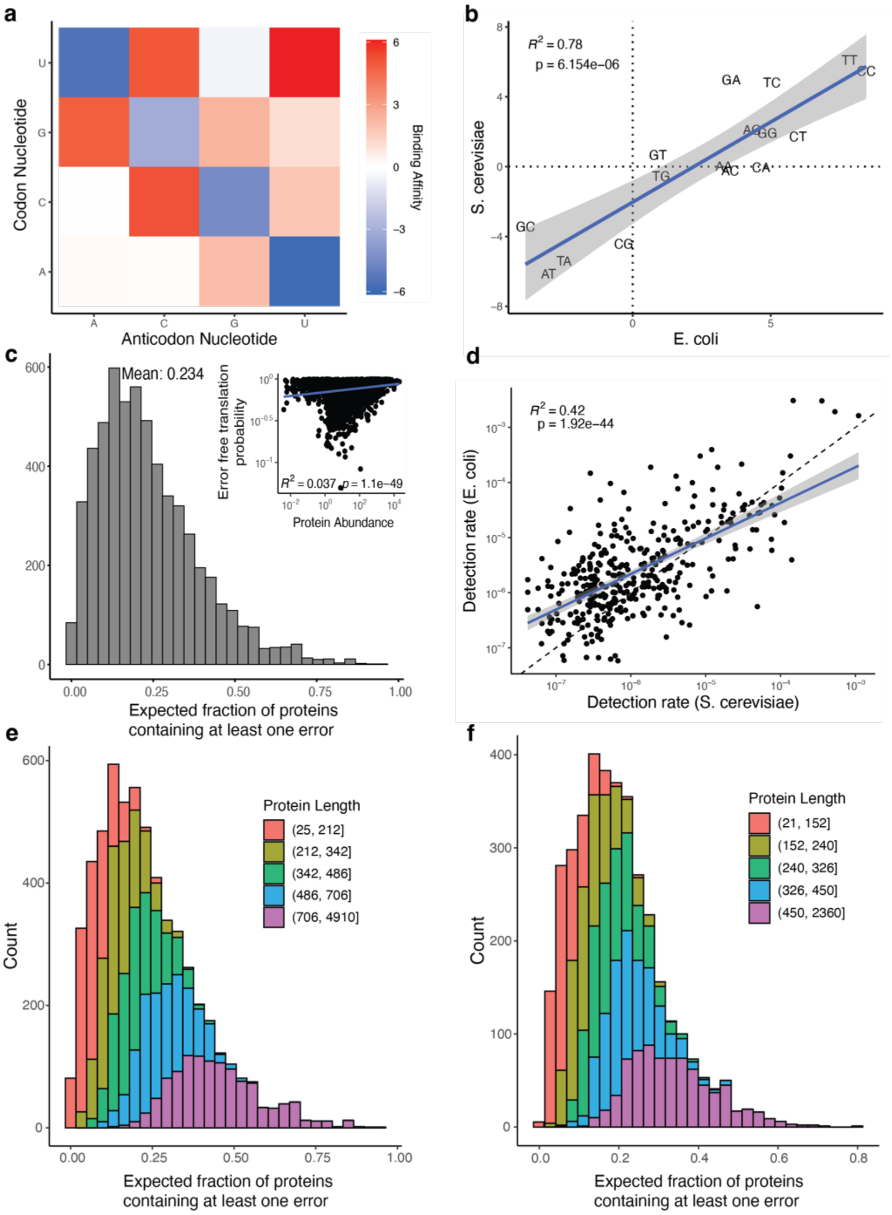

Figure S7: a) Posterior mean of effective binding affinities of individual nucleotides in the codon and anticodon. Watson-Crick codon/anticodon pairs show high effective binding affinity (blue). **b)** Comparison of the effective binding affinities of *E. coli* and *S. cerevisiae*. In addition to a strong correlation, most parameters are in the first and second quadrant, thus sharing their affinity tendency. **c)** Expected fraction of proteins with at least one amino acid misincorporation. The mean fraction of 0.234 indicates that close to a quarter of all *S. cerevisiae* proteins produced are expected to contain at least one amino acid misincorporation. **d)** Comparison of the individual codon to amino acid misincorporation rates (detection rates, e.g, AAC to V, or CAU to Q) between *E. coli* and *S. cerevisiae*. Error detection rates tend to be higher in *E. coli* when error rates are low. **e, f)** stacked histogram of expected fraction of proteins with at least one amino acid misincorporation for *S. cerevisiae* (e) and *E. coli* (f), respectively. Proteins are binned based on length. Longer proteins have a higher chance to contain at least on error.

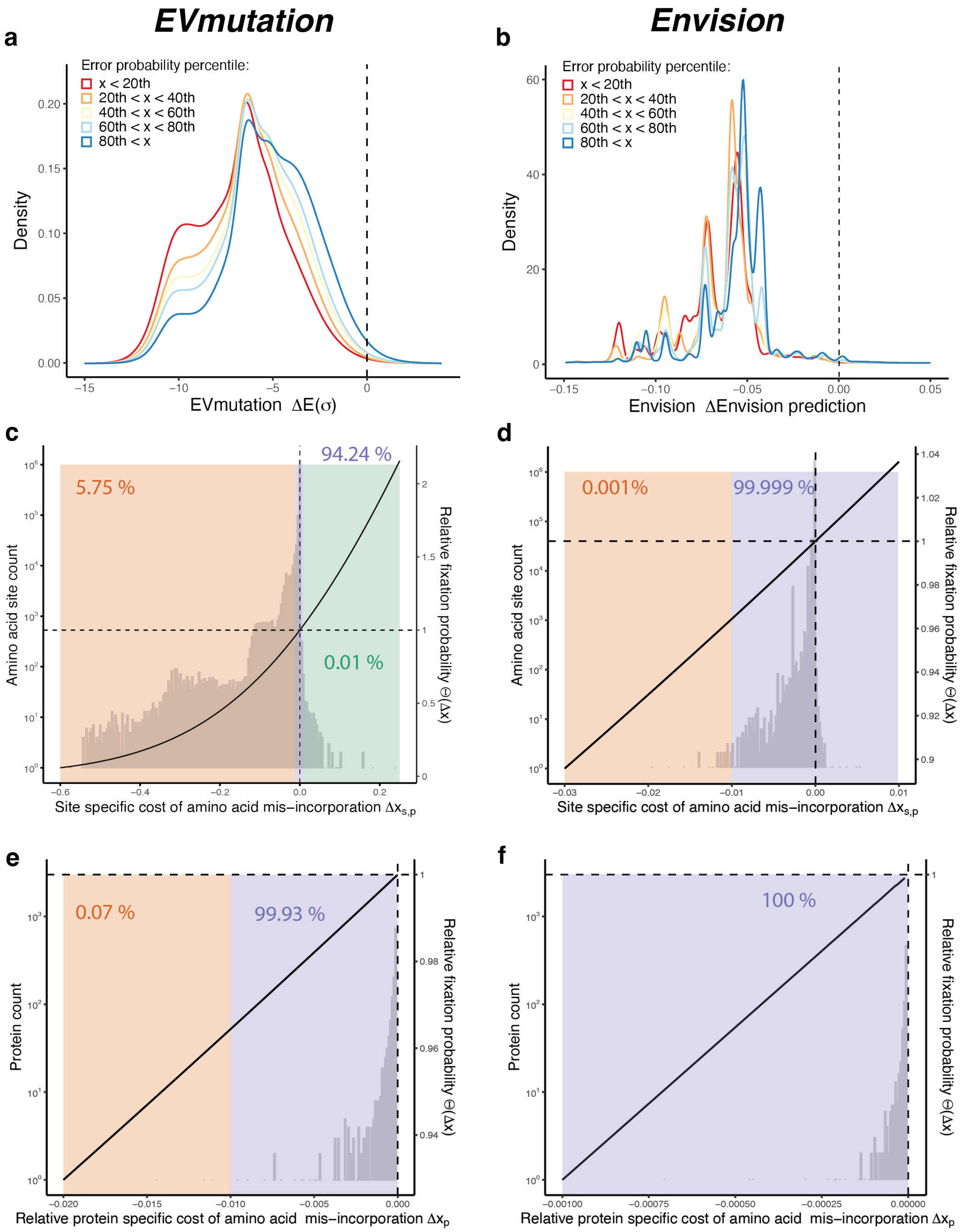

Figure S8: The estimated evolutionary impact of amino acid misincorporation is similar for EVmutation and Envision based fitness estimates. a, b) More deleterious substitutions have lower error probability in *S. cerevisiae* (error probability decreases from light to dark blue) based on fitness estimates obtained with **a)** EVmutations and **b)** Envision. **c, d)** Distribution of site-specific costs of amino acid misincorporations estimated with **c)** EVmutation and **d)** Envision. Using Envision, no amino misincorporations are considered advantageous. **e, f)** Distribution of protein-specific cost of amino acid misincorporations according to **e)** EVmutation and **f)** Envision. Fitness burden was calculated as the sum of individual site contributions and weighted by the relative contribution of a protein to the proteome based on protein abundance.

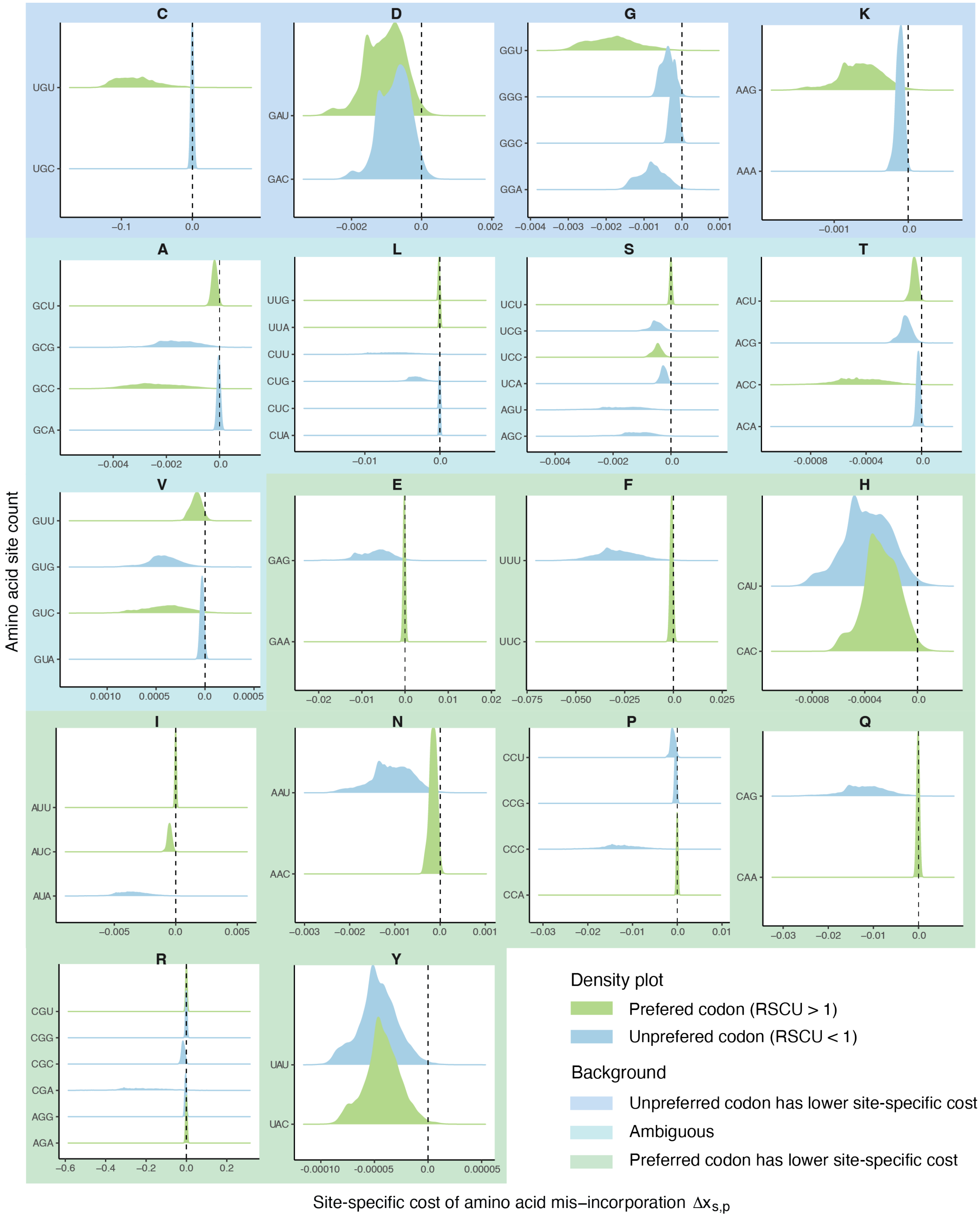

Figure S9: Distributions of site-specific costs of amino acid misincorporations differs between synonymous codons in *S. cerevisiae*. Amino acids are grouped based on the differences in the distribution of site-specific cost of amino acid misincorporations of synonymous codons.

Distribution color indicates if a codon is preferentially used (RSCU > 1, green) or not (RSCU < 1, blue). In contrast to *E. coli,* where preferred codons for two codon amino acids reduce the impacts of amino acid misincorporations, this is not the case here, in *S. cerevisiae* (for example, in the cases of Cysteine (C) and Lysine (K)). P-values representing the comparison of the distributions indicate a significant difference for all distributions in *S. cerevisiae* (Table S4).

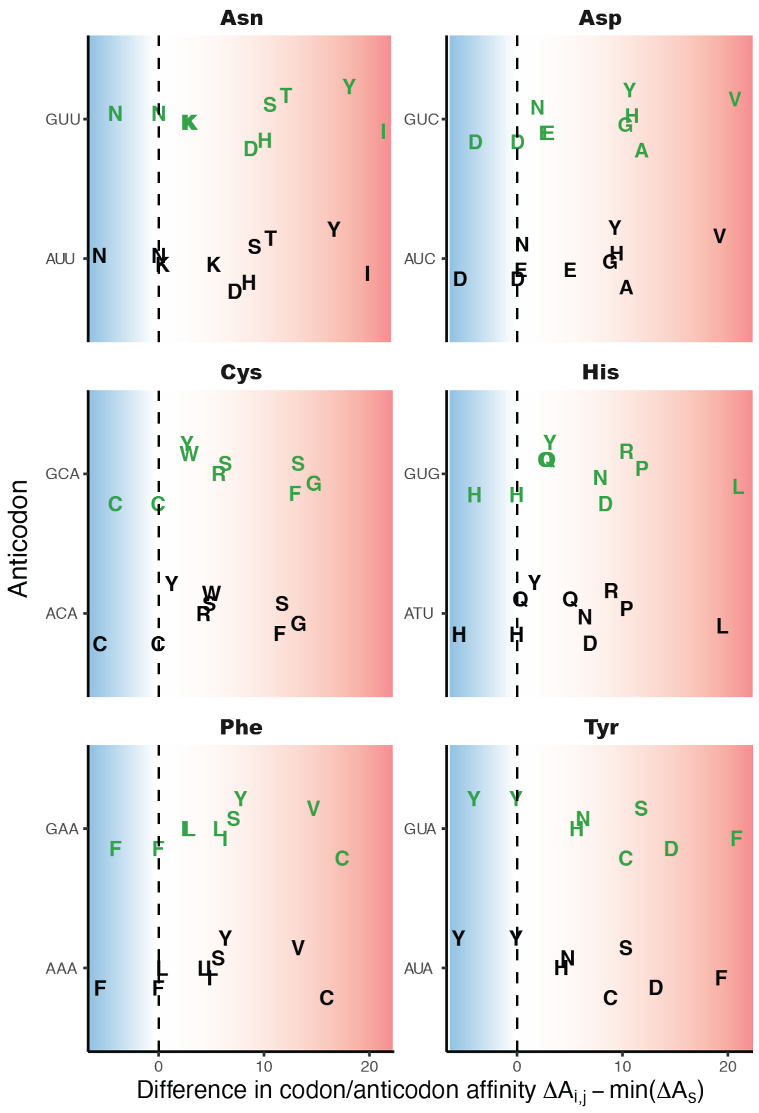

Figure S10: Minimizing tRNA misbinding by tRNA choice in *S. cerevisiae*. Impact of the tRNA pool on translational errors. Difference in effective codon/anticodon binding affinity for interactions with up to one codon/anticodon mismatch, for all two-codon amino acids with only one tRNA present in *S. cerevisiae*. The ability of the ribosome to discriminate between correct and incorrect binding events is diminished if we assume that the missing tRNA (black) would be present rather than the existing tRNA (naturally present, green).

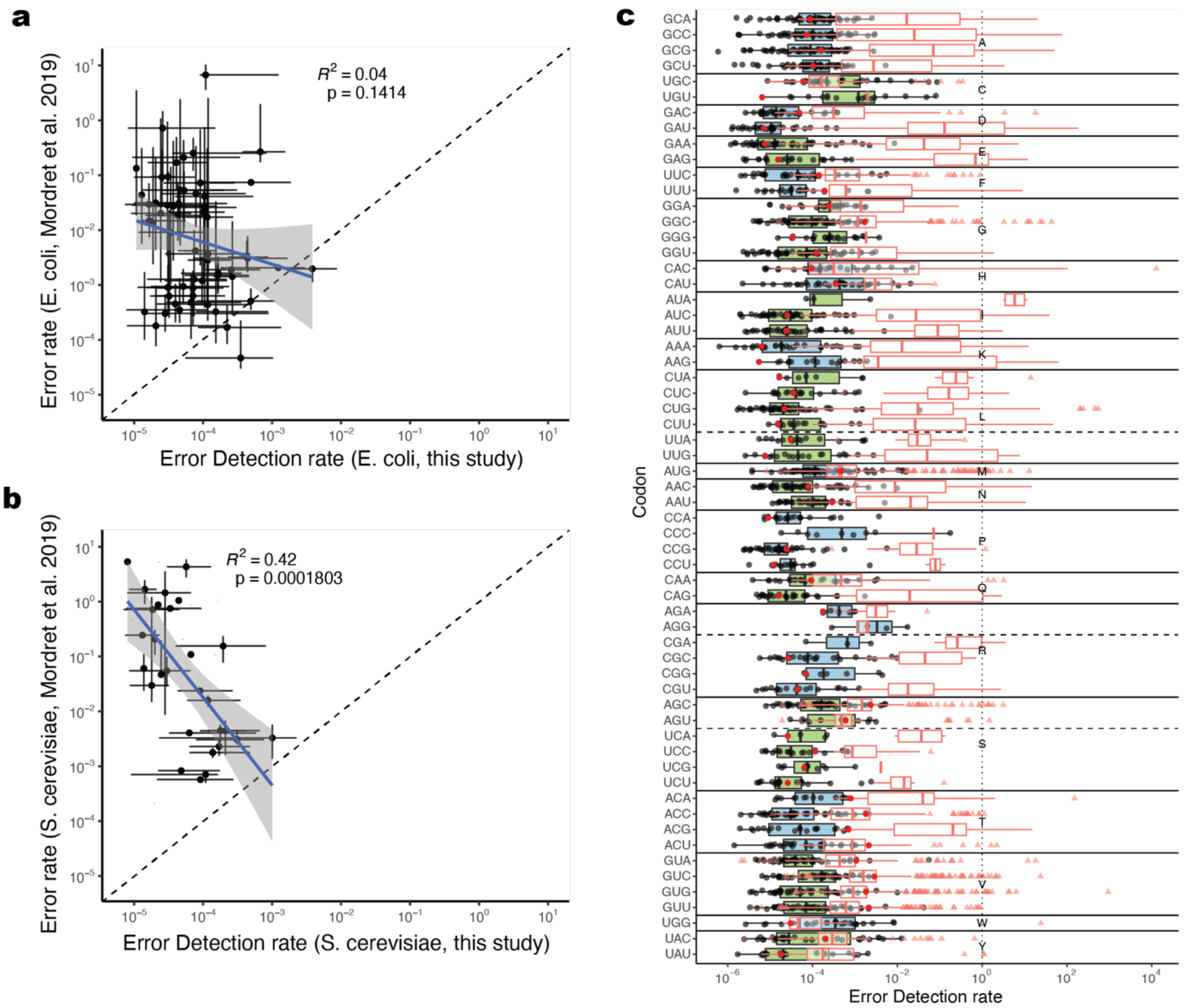

Figure S11: Comparison of error rate estimates. a, b) Comparison of the error rates estimated in (Mordret et al. 2019) with the estimates from this study for **a)** *E. coli* and **b)** *S. cerevisiae*. Error rates estimated in this study are generally lower in both organisms. **c)** Codon specific error detection rates in *E. coli* (thick black line indicates median, box indicates 25th and 75th percentiles, whiskers indicate 1.5 times the inter-quartile range). For reference, red dot indicates our detection rate for the *E. coli* data used by Mordret et al. (2019). Red bar graph shows error rates estimated in Mordret et al. (2019).

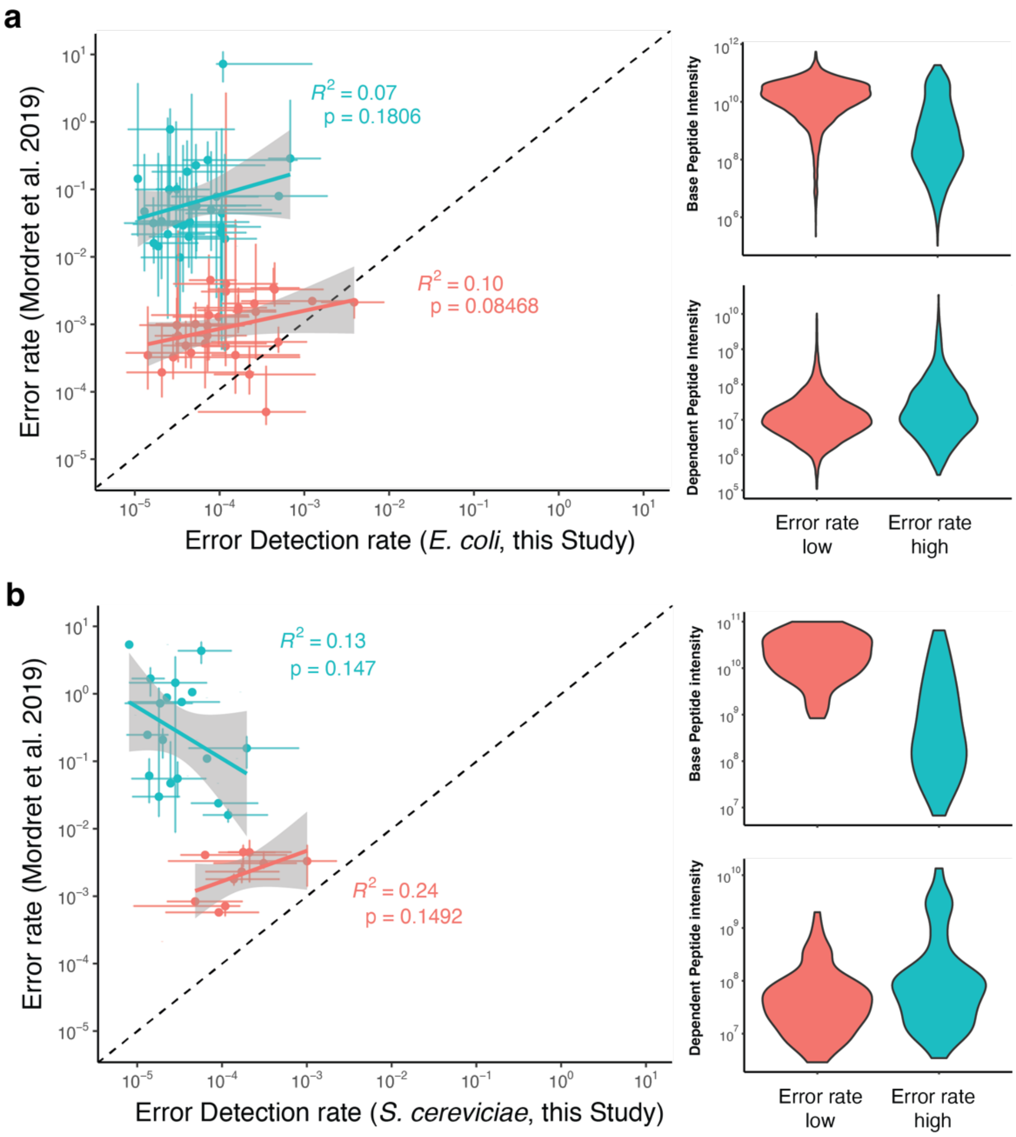

Figure S12: Comparison of the error rates estimated in Mordret et al. (2019) with the estimates from this study for **a)** *E. coli* and **b)** *S. cerevisiae*. Data was split in two groups based on the mean error rate estimated by Mordret et al. (2019); blue: $Error\ rate > 3.5 \times 10^{-3}$ (Error rate high), red: $Error\ rate < 3.5 \times 10^{-3}$ (Error rate low). Violin plots show the intensities of the base and dependent peptide in the two groups. The large difference in base peptide intensity indicates a large noise component in the error rate estimates of the blue group due to the low base peptide intensity (Carrillo et al. 2010). Overall, the base peptide intensities are lower, and since these are in the denominator (the error rate is calculated as the ratio of the dependent and the base peptide intensities from Mordret at al. 2019), this could lead to inflation of the error rates (indeed the blue points deviate in the upper left quadrant). These error rates estimated previously are close to and even above 1 (meaning errors are more likely than correct amino acid). The error estimates that correspond to high intensity peaks (shown in red) give a much better correlation with our estimates in *S. cerevisiae* (R^2=0.24), but not in E. coli (R^2=0.10) compared to Figure S11a and b.

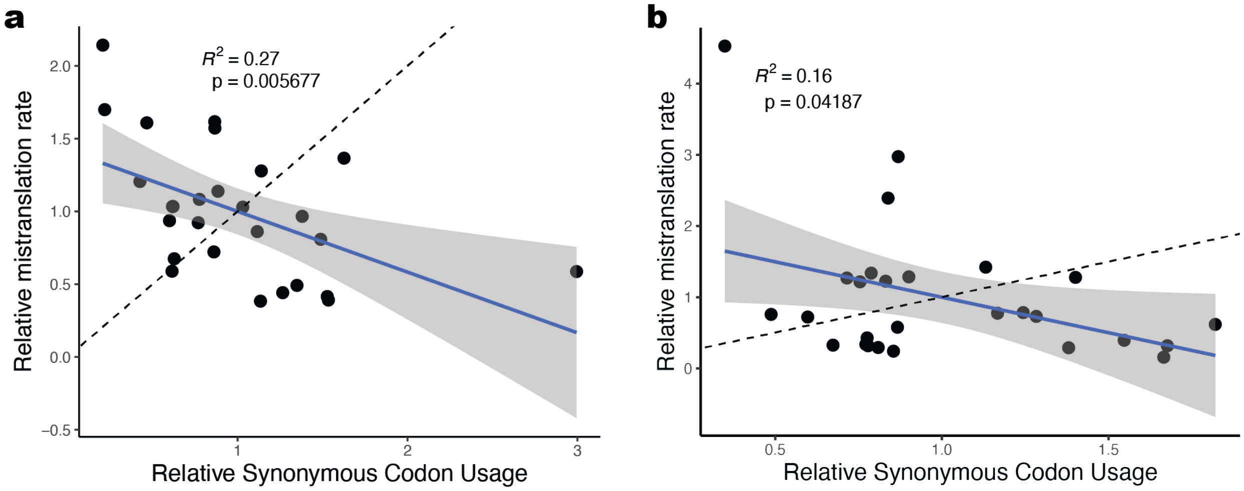

Figure 13: Codon usage does not reflect mistranslation rate. a, b) Codons usage, represented as relative synonymous codon usage was compared with the relative mistranslation rate estimated in this study, following (Sun and Zhang 2022) for **a)** *E. coli* and **b)** *S. cerevisiae*. In agreement with Sun and Zhang (2022), we found a weak negative correlation.

Supplementary Data & Tables

Data S1: Summary of *E. coli* and *S. cerevisiae* datasets explored in this study and the number of amino acid misincorporations and peptides detected in each dataset (see Data_S1_datasets.xslx).

Data S2: Summary of codon specific error detection rates for *E. coli* and *S. cerevisiae* (see Data_S2_error_detection_rates.xlsx).

Data S3: Summary of PTMs that mask potential amino acid misincorporations (see Data_S3_ptm_masking.csv).

Data S4: Detected amino acid misincorporations, peptide and codon count for each dataset that passed all quality metrics.

Table S1: mTEL model parameters estimated for *E. coli* and *S. cerevisiae*. The *E. coli* model parameters include interaction with the Lysidine modified nucleotide (denoted as L) in the anticodon used to decode AUA.

	**E. coli**			**S. cerevisiae**		
**Parameter**	**Posterior mean**	**5%**	**95%**	**Posterior mean**	**5%**	**95%**
**AA**	3.214726	3.176039	3.272070	0.236423	0.1451952	0.3958902
**AC**	3.748255	3.702175	3.800652	0.125760	0.0305024	0.2894249
**AG**	4.411252	4.374065	4.468128	2.161263	2.0400825	2.3426570
**CA**	4. 563604	4.525120	4.610314	-0.095805	-0.195542	0.0016996
**CC**	8.467473	8.417083	8.528389	5.272105	5.1527597	5.385575
**CT**	6.019738	5.976320	6.064627	1.810633	1.7087638	1.910929
**TC**	5.372364	5.350539	5.401506	5.143619	5.0431567	5.260081
**TT**	8.116570	8.086854	8.158565	6.266714	6.1709223	6.378583
**TG**	1.054184	1.033891	1.079291	-0.533649	-0.6316556	-0.427040
**GA**	3.283392	3.253748	3.332301	4.973449	4.9093120	5.063151
**GT**	0.907996	0.886118	0.940231	1.077082	1.0215029	1.168301
**GG**	5.132492	5.108529	5.168441	2.420570	2.3682179	2.512241
**AT**	-3.200599	-3.234803	-3.154938	-6.031697	-6.1229576	-5.877032
**TA**	-2.638884	-2.663381	-2.613332	-5.612437	-5.7103089	-5.499516
**GC**	-4.155617	-4.174342	-4.124052	-3.227225	-3.2784262	-3.142651
**CG**	-0.612063	-0.652283	-0.570131	-4.584306	-4.6858969	-4.487009
**AL**	-8.186579	-8.296169	-8.101169			
**CL**	-4.894885	-4.937699	-4.830650			
**TL**	-4.657662	-4.686112	-4.588831			
**GL**	-1.421939	-1.452926	-1.378731			
**Pos1**	2.213301	2.209546	2.218894	1.777494	1.774573	1.782323
**Pos2**	1.558439	1.555680	1.562240	2.034535	2.030993	2.040044

Table S2: Summary of fitness effects of amino acid substitutions binned by their associated error probability (to occur as amino acid misincorporation). Less likely amino acid misincorporations show a more deleterious fitness effect in both *E. coli* and *S. cerevisiae,* as well as for estimates obtained from EVmutation and Envision.

		EVmutation		Envision	
	bin	mean fitness effect	median fitness effect	mean fitness effect	median fitness effect
S. cerevisiae	x < 20th	-6.82	-6.63	-0.0751	-0.0646
	20th < x < 40th	-6.35	-6.24	-0.0709	-0.0601
	40th < x < 60th	-6.01	-5.92	-0.0690	-0.0586
	60th < x < 80th	-5.77	-5.71	-0.0643	-0.0568
	80th < x	-5.15	-5.13	-0.0592	-0.0529
E. coli	x < 20th	-7.52	-7.49		
	20th < x < 40th	-6.95	-6.91		
	40th < x < 60th	-6.68	-6.61		
	60th < x < 80th	-6.28	-6.24		
	80th < x	-6.01	-5.95		

Table S3: Error rate comparison of selected codons with biochemical error rate estimates. Error rates for Mordret et al. 2019 were recalculated with correct denominator. (*) Mean value; ([1]) E. coli; ([2]) S. cerevisiae

	Proteome wide studies			
**Codon**	**eTEL***	**mTEL**	**Mordret et al. 2019***	**Biochemical studies**
AAC[1]	4.34×10^{-4}	3.73×10^{-4}	1.28×10^{-1}	3.8×10^{-4} (Kramer and Farabaugh 2007)<br>1.8×10^{-4} (Parker et al. 1983)<br>$5-6 \times 10^{-3}$ (Khazaie et al. 1984)
AAU[1]	2.75×10^{-4}	3.61×10^{-4}	5.35×10^{-2}	1.6×10^{-3} (Kramer and Farabaugh 2007)<br>1.5×10^{-3} (Parker et al. 1983)<br>$5-6 \times 10^{-3}$ (Khazaie et al. 1984)<br>5×10^{-3} (Parker and Holtz 1984)
AGA[1]	5.37×10^{-4}	5.19×10^{-4}	7.8×10^{-3}	3.6×10^{-3} (Kramer and Farabaugh 2007)
AGG[1]	6.28×10^{-3}	1.28×10^{-5}	1.67×10^{-3}	3.1×10^{-3} (Kramer and Farabaugh 2007)
AUA[1]	8.26×10^{-4}	6.42×10^{-6}	8.42×10^{-1}	3.5×10^{-4} (Kramer and Farabaugh 2007)
CGC[1]	1.05×10^{-3}	4.58×10^{-3}	1.25×10^{-1}	2×10^{-4} (Edelmann and Gallant 1977)
CGU[1]	1.75×10^{-4}	3.44×10^{-7}	6.52×10^{-2}	2×10^{-4} (Edelmann and Gallant 1977)<br>1.3×10^{-3} (Bouadloun et al. 1983)<br>$1.5-7 \times 10^{-3}$ (Laughrea et al. 1987)
GGC[1]	2.35×10^{-4}	9.23×10^{-6}	1.46×10^{-2}	1×10^{-3} (Toth et al. 1988)
UGG[1]	1.17×10^{-3}	9.59×10^{-6}	8.74×10^{-2}	4×10^{-3} (Bouadloun et al. 1983)
GCU[2]	3.56×10^{-4}	3.99×10^{-5}	2.22×10^{-3}	2×10^{-5} (Stansfield et al. 1998)
UAC[2]	5.55×10^{-5}	7.3×10^{-6}	N/A	5×10^{-6} (Stansfield et al. 1998)

Table S4: Comparison of the distributions in Figure 4 (*E. coli*) and Figure S9 (*S. cer*). P-values are based on a two-sided Wilcoxon test.

E. coli

**Amino Acid**				
**one letter**	**three letter**	**Codon 1**	**Codon 2**	**P-value**
A	Ala	GCU	GCC	2.7e-11
			GCA	0
			GCG	0
		GCC	GCA	0
			GCG	0
		GCA	GCG	0
C	Cys	UGU	UGC	0
D	Asp	GAU	GAC	0
E	Glu	GAA	GAG	0
F	Phe	UUU	UUC	0
G	Gly	GGU	GGC	0
			GGA	0
			GGG	0
		GGC	GGA	0
			GGG	0
		GGA	GGG	0
H	His	CAU	CAC	0
I	Ile	AUU	AUC	0
			AUA	0
		AUC	AUA	0
K	Lys	AAA	AAG	0
L	Leu	UUA	UUG	0
			CUU	0
			CUC	0
			CUA	0
			CUG	0
		UUG	CUU	0
			CUC	0
			CUA	0
			CUG	0
		CUU	CUC	0
			CUA	0
			CUG	0
		CUC	CUA	0
			CUG	1.14e-23
		CUA	CUG	0
N	Asn	AAU	AAC	0
P	Pro	CCU	CCC	0
			CCA	0
			CCG	0
		CCC	CCA	0
			CCG	0
		CCA	CCG	0
Q	Gln	CAA	CAG	0
R	Arg	AGA	AGG	0
			CGU	0
			CGC	0
			CGA	0
			CGG	0
		AGG	CGU	0
			CGC	0
			CGA	0
			CGG	0
		CGU	CGC	0
			CGA	0
			CGG	0
		CGC	CGA	0
			CGG	0
		CGA	CGG	0

S	Ser	AGU	AGC	0
			UCU	0
			UCC	0
			UCA	0
			UCG	0
		AGC	UCU	0
			UCC	0
			UCA	0
			UCG	0
		UCU	UCC	0
			UCA	0
			UCG	0
		UCC	UCA	0
			UCG	0
		UCA	UCG	0
T	Thr	ACU	ACC	0
			ACA	0
			ACG	0
		ACC	ACA	0
			ACG	0
		ACA	ACG	0
V	Val	GUU	GUC	0
			GUA	0
			GUG	0
		GUC	GUA	0
			GUG	0
		GUA	GUG	0
Y	Tyr	UAU	UAC	0.5675

S. cerevisiae

**Amino Acid**				
**one letter**	**three letter**	**Codon 1**	**Codon 2**	**P-value**
A	Ala	GCU	GCC	0
			GCA	0
			GCG	0
		GCC	GCA	0
			GCG	0
		GCA	GCG	0
C	Cys	UGU	UGC	0
D	Asp	GAU	GAC	0
E	Glu	GAA	GAG	0
F	Phe	UUU	UUC	0
G	Gly	GGU	GGC	0
			GGA	0
			GGG	0
		GGC	GGA	0
			GGG	0
		GGA	GGG	0
H	His	CAU	CAC	0
I	Ile	AUU	AUC	0
			AUA	0
		AUC	AUA	0
K	Lys	AAA	AAG	0
L	Leu	UUA	UUG	0
			CUU	0
			CUC	0
			CUA	0
			CUG	0
		UUG	CUU	0
			CUC	0
			CUA	0
			CUG	0
		CUU	CUC	0
			CUA	0
			CUG	0

		CUC	CUA	0
			CUG	0
		CUA	CUG	0
N	Asn	AAU	AAC	0
P	Pro	CCU	CCC	0
			CCA	0
			CCG	0
		CCC	CCA	0
			CCG	0
		CCA	CCG	0
Q	Gln	CAA	CAG	0
R	Arg	AGA	AGG	0
			CGU	0
			CGC	0
			CGA	0
			CGG	0
		AGG	CGU	0
			CGC	0
			CGA	0
			CGG	0
		CGU	CGC	0
			CGA	0
			CGG	0
		CGC	CGA	0
			CGG	0
		CGA	CGG	0
S	Ser	AGU	AGC	0
			UCU	0
			UCC	0
			UCA	0
			UCG	0
		AGC	UCU	0
			UCG	0
		UCU	UCC	0
			UCA	0
			UCG	0
		UCC	UCA	0
			UCG	0
		UCA	UCG	0
T	Thr	ACU	ACC	0
			ACA	0
			ACG	0
		ACC	ACA	0
			ACG	0
		ACA	ACG	0
V	Val	GUU	GUC	0
			GUA	0
			GUG	0
		GUC	GUA	0
			GUG	5.33e-23
		GUA	GUG	0
Y	Tyr	UAU	UAC	0

Supplementary References

Bouadloun F, Donner D, Kurland CG. 1983. Codon-specific missense errors in vivo. *EMBO J.* 2:1351–1356.

Carrillo B, Yanofsky C, Laboissiere S, Nadon R, Kearney RE. 2010. Methods for combining peptide intensities to estimate relative protein abundance. *Bioinformatics* 26:98–103.

Chang H-Y, Kong AT, da Veiga Leprevost F, Avtonomov DM, Haynes SE, Nesvizhskii AI. 2020. Crystal-C: A Computational Tool for Refinement of Open Search Results. *J. Proteome Res.* 19:2511–2515.

Edelmann P, Gallant J. 1977. Mistranslation in E. coli. *Cell* 10:131–137.

Fluitt A, Pienaar E, Viljoen H. 2007. Ribosome kinetics and aa-tRNA competition determine rate and fidelity of peptide synthesis. *Comput. Biol. Chem.* 31:335–346.

Geiszler DJ, Kong AT, Avtonomov DM, Yu F, Leprevost F da V, Nesvizhskii AI. 2021. PTM-Shepherd: Analysis and Summarization of Post-Translational and Chemical Modifications From Open Search Results. *Mol. Cell. Proteomics* 20:100018.

Gilchrist MA. 2007. Combining models of protein translation and population genetics to predict protein production rates from codon usage patterns. *Mol. Biol. Evol.* 24:2362–2372.

Hopf TA, Green AG, Schubert B, Mersmann S, Schärfe CPI, Ingraham JB, Toth-Petroczy A, Brock K, Riesselman AJ, Palmedo P, et al. 2018. The EVcouplings Python framework for coevolutionary sequence analysis. *Bioinformatics* 35:1582–1584.

Hopf TA, Ingraham JB, Poelwijk FJ, Schärfe CPI, Springer M, Sander C, Marks DS. 2017. Mutation effects predicted from sequence co-variation. *Nat. Biotechnol.* 35:128–135.

Jones P, Côté R. 2008. The PRIDE Proteomics Identifications Database: Data Submission, Query, and Dataset Comparison. In: Thompson JD, Ueffing M, Schaeffer-Reiss C, editors. Functional Proteomics: Methods and Protocols. Totowa, NJ: Humana Press. p. 287–303.

Khazaie K, Buchanan JH, Rosenberger RF. 1984. The accuracy of Q beta RNA translation. 1. Errors during the synthesis of Q beta proteins by intact Escherichia coli cells. *Eur. J. Biochem.* 144:485–489.

Kong AT, Leprevost FV, Avtonomov DM, Mellacheruvu D, Nesvizhskii AI. 2017. MSFragger: ultrafast and comprehensive peptide identification in mass spectrometry–based proteomics. *Nat. Methods* 14:513–520.

Kramer EB, Farabaugh PJ. 2007. The frequency of translational misreading errors in E. coli is largely determined by tRNA competition. *RNA* 13:87–96.

Larson MH, Mooney RA, Peters JM, Windgassen T, Nayak D, Gross CA, Block SM, Greenleaf WJ, Landick R, Weissman JS. 2014. A pause sequence enriched at translation start sites drives transcription dynamics in vivo. *Science* 344:1042–1047.

Laughrea M, Latulippe J, Filion AM, Boulet L. 1987. Mistranslation in twelve Escherichia coli ribosomal proteins. Cysteine misincorporation at neutral amino acid residues other than tryptophan. *Eur. J. Biochem.* 169:59–64.

Luppino F, Adzhubei IA, Cassa CA, Toth-Petroczy A. 2023. DeMAG predicts the effects of variants in clinically actionable genes by integrating structural and evolutionary epistatic features. *Nat. Commun.* 14:1–14.

Lynch M. 2010. Evolution of the mutation rate. *Trends Genet.* 26:345–352.

Mordret E, Dahan O, Asraf O, Rak R, Yehonadav A, Barnabas GD, Cox J, Geiger T, Lindner AB, Pilpel Y. 2019. Systematic Detection of Amino Acid Substitutions in Proteomes Reveals Mechanistic Basis of Ribosome Errors and Selection for Translation Fidelity. *Mol. Cell* 75:427-441.e5.

Parker J, Holtz G. 1984. Control of basal-level codon misreading in Escherichia coli. *Biochem. Biophys. Res. Commun.* 121:487–492.

Parker J, Johnston TC, Borgia PT, Holtz G, Remaut E, Fiers W. 1983. Codon usage and mistranslation. In vivo basal level misreading of the MS2 coat protein message. *J. Biol. Chem.* 258:10007–10012.

Perez-Riverol Y, Bai J, Bandla C, García-Seisdedos D, Hewapathirana S, Kamatchinathan S, Kundu DJ, Prakash A, Frericks-Zipper A, Eisenacher M, et al. 2022. The PRIDE database resources in 2022: a hub for mass spectrometry-based proteomics evidences. *Nucleic Acids Res.* 50:D543–D552.

Perez-Riverol Y, Csordas A, Bai J, Bernal-Llinares M, Hewapathirana S, Kundu DJ, Inuganti A, Griss J, Mayer G, Eisenacher M, et al. 2018. The PRIDE database and related tools and resources in 2019: improving support for quantification data. *Nucleic Acids Res.* 47:D442–D450.

Sella G, Hirsh AE. 2005. The application of statistical physics to evolutionary biology. *Proc. Natl. Acad. Sci. U. S. A.* 102:9541–9546.

Stansfield I, Jones KM, Herbert P, Lewendon A, Shaw WV, Tuite MF. 1998. Missense translation errors in Saccharomyces cerevisiae. *J. Mol. Biol.* 282:13–24.

Sun M, Zhang J. 2022. Preferred synonymous codons are translated more accurately: Proteomic evidence, among-species variation, and mechanistic basis. *Science Advances* 8:eabl9812.

Toth MJ, Murgola EJ, Schimmel P. 1988. Evidence for a unique first position codon-anticodon mismatch in vivo. *J. Mol. Biol.* 201:451–454.

Tsai IJ, Bensasson D, Burt A, Koufopanou V. 2008. Population genomics of the wild yeast *Saccharomyces paradoxus*: Quantifying the life cycle. *Proceedings of the National Academy of Sciences* 105:4957–4962.

da Veiga Leprevost F, Haynes SE, Avtonomov DM, Chang H-Y, Shanmugam AK, Mellacheruvu D, Kong AT, Nesvizhskii AI. 2020. Philosopher: a versatile toolkit for shotgun proteomics data analysis. *Nat. Methods* 17:869–870.

Wang M, Herrmann CJ, Simonovic M, Szklarczyk D, von Mering C. 2015. Version 4.0 of PaxDb: Protein abundance data, integrated across model organisms, tissues, and cell-lines. *Proteomics* 15:3163–3168.

Weinberg DE, Shah P, Eichhorn SW, Hussmann JA, Plotkin JB, Bartel DP. 2016. Improved Ribosome-Footprint and mRNA Measurements Provide Insights into Dynamics and Regulation of Yeast Translation. *Cell Rep.* 14:1787–1799.

Yu F, Teo GC, Kong AT, Haynes SE, Avtonomov DM, Geiszler DJ, Nesvizhskii AI. 2020. Identification of modified peptides using localization-aware open search. *Nature Communications* [Internet] 11. Available from: http://dx.doi.org/10.1038/s41467-020-17921-y
